# *Mycobacterium tuberculosis* encodes a YhhN family membrane protein with lysoplasmalogenase activity that protects against toxic host lysolipids

**DOI:** 10.1016/j.jbc.2022.101849

**Published:** 2022-03-18

**Authors:** Marianne S. Jurkowitz, Abul K. Azad, Paula C. Monsma, Tracy L. Keiser, Jean Kanyo, TuKiet T. Lam, Charles E. Bell, Larry S. Schlesinger

**Affiliations:** 1 Department of Biological Chemistry and Pharmacology, The Ohio State University, Columbus, Ohio, USA; 2 Host Pathogen Interactions Program, Texas Biomedical Research Institute, San Antonio, Texas, USA; 3 Department of Neuroscience, Ohio State University, Columbus, Ohio, USA; 4 Department of Moleculaire Microbiologie, Vrije Universiteit, Amsterdam, the Netherlands; 5 Keck MS & Proteomics Resource, Yale University, New Haven, Connecticut, USA; 6 Department of Molecular Biophysics and Biochemistry, Yale University, New Haven, Connecticut, USA; 7 Department of Chemistry and Biochemistry, The Ohio State University, Columbus, Ohio, USA

**Keywords:** *Mycobacterium tuberculosis* Rv1401 gene, *Mycobacterium smegmatis*, lysoplasmalogenase, YhhN protein, macrophage, phospholipase A, lysoplasmalogen/lysophospholipid, cell injury and protection, spheroplasts, cell wall–deficient, aa, amino acids, DDM, dodecylmaltoside, GPC, glycerophosphocholine, GPCR, GTP-protein coupled receptor, GPE, glycerophosphoethanolamine, LPA, lysophosphatidic acid (1-acyl-*sn*-glycerol-3-phosphate), MDM, monocyte-derived macrophage, *M.smeg*, *Mycobacterium smegmatis*, *M.tb*, *Mycobacterium tuberculosis*, MtbYhhN, protein product of *M. tuberculosis* gene Rv1401, *ORF*, open reading frame, PG, peptidoglycan, pLPC, lysoplasmenylcholine, 1-alkenyl-*sn*-glycero-3-phosphocholine, pLPE, lysoplasmenylethanolamine, 1-alkenyl-*sn*-glycero-3-phosphoethanolamine, PLA_2_, phospholipase A_2_, *RBC*, red blood cell, *TB*, tuberculosis, TEV, tobacco etch virus, VC, vector control

## Abstract

The pathogen *Mycobacterium tuberculosis* (*M.tb*) resides in human macrophages, wherein it exploits host lipids for survival. However, little is known about the interaction between *M.tb* and macrophage plasmalogens, a subclass of glycerophospholipids with a vinyl ether bond at the *sn-1* position of the glycerol backbone. Lysoplasmalogens, produced from plasmalogens by hydrolysis at the *sn-2* carbon by phospholipase A_2_, are potentially toxic but can be broken down by host lysoplasmalogenase, an integral membrane protein of the YhhN family that hydrolyzes the vinyl ether bond to release a fatty aldehyde and glycerophospho-ethanolamine or glycerophospho-choline. Curiously, *M.tb* encodes its own YhhN protein (MtbYhhN), despite having no endogenous plasmalogens. To understand the purpose of this protein, the gene for MtbYhhN (Rv1401) was cloned and expressed in *Mycobacterium smegmatis* (*M.smeg*). We found the partially purified protein exhibited abundant lysoplasmalogenase activity specific for lysoplasmenylethanolamine or lysoplasmenylcholine (pLPC) (V_max_∼15.5 μmol/min/mg; K_m_∼83 μM). Based on cell density, we determined that lysoplasmenylethanolamine, pLPC, lysophosphatidylcholine, and lysophosphatidylethanolamine were not toxic to *M.smeg* cells, but pLPC and LPC were highly toxic to *M.smeg* spheroplasts, which are cell wall–deficient mycobacterial forms. Importantly, spheroplasts prepared from *M.smeg* cells overexpressing MtbYhhN were protected from membrane disruption/lysis by pLPC, which was rapidly depleted from the media. Finally, we found that overexpression of full-length MtbYhhN in *M.smeg* increased its survival within human macrophages by 2.6-fold compared to vector controls. These data support the hypothesis that MtbYhhN protein confers a growth advantage for mycobacteria in macrophages by cleaving toxic host pLPC into potentially energy-producing products.

Tuberculosis (TB), caused by the intracellular bacterial pathogen *Mycobacterium tuberculosis* (*M*.*tb*), is a leading cause of infectious disease mortality worldwide. Replication of *M.tb* within host macrophages is critical to establish infection. Once inside the macrophage, *M.tb* lives within membrane-bound phagosomal vacuoles. Mtb ensures its continued survival through modulating properties of its phagosome that facilitate fusion with the early endosomal compartment, blocks fusion with lysosomes, and ensures only a mildly acidified environment (pH 6.0 to pH 6.8) (1, 2, 3, 4). Within the phagosome, *M.tb* accesses nutrients and can use host lipids as energy sources for survival (5, 6, 7).

*M.tb* has a unique cell envelope structure that includes the cell wall and the cytoplasmic membrane. The wall is composed of an outer membrane including the acyl chains of mycolic acids that are covalently attached to the arabinogalactan (AG) layer. The AG is covalently linked to the peptidoglycan (PG) layer, which is essential for cell integrity, maintenance of the rod shape, and virulence (8). The cell wall has low permeability to many molecules, nutrients, and antimicrobials. The PG layer is dynamic and is continually being reformed and remodeled *via* reactions taking place in part within the cytoplasmic membrane that lies beneath the PG layer.

The cytoplasmic membrane of *M.tb* is a bilayer that is largely composed of the phospholipids phosphatidylethanolamine, cardiolipin, and phosphatidylinositol mannosides (9). It is similar to mammalian cell membranes including the mitochondrial inner membrane.

In mycobacteria, disruption of PG synthesis and/or digestion of the PG layer by hydrolases leads to formation of cell wall–deficient forms called spheroplasts or L-form bacilli. These cell forms have a complete cytoplasmic membrane but an incomplete or defective cell wall and display a variety of shapes and morphologies (10, 11). Spheroplasts contain cytoplasm, endoplasmic reticulum, nucleic acids, and enzymes needed for energy production and protein synthesis (10). They are osmotically fragile and require an isosmotic suspension media to prevent swelling. Spheroplasts are living and if placed in a nurturing media can regenerate their thick outer wall, regain the rod shape of normal cells, and divide and grow in solution or form colonies on agar (11, 12). Spheroplasts are commonly prepared *in vitro* (13) and are used in the research reported here.

Little is known about the conditions required for the formation of mycobacteria spheroplasts *in vivo* and their significance in TB or other diseases. It is hypothesized that the imbalance of the cell’s ability to synthesize and degrade its thick outer wall results in cell wall deficiency (14). Under unfavorable conditions where mycobacteria are exposed to a damaging environment and host defense mechanism, they are capable of forming spheroplasts (14, 15). This could facilitate bacterial survival and persistence under unfavorable conditions in the host, potentially underlying latent TB (14).

Macrophages play a decisive role in host responses to intracellular *M.tb* (1, 2). Plasmalogens are components of the cell membranes of macrophages and alveolar epithelial cells, while *M.tb* and all other aerobic bacteria have no plasmalogens (16, 17). Plasmalogens are glycerophospholipids that contain a cis-vinyl ether bond at the *sn*-1 position of the glycerol backbone, as well as enrichment of polyunsaturated fatty acids such as arachidonic acid at the *sn*-2 carbon (18, 19). They are critical components of mammalian cell membranes, where they affect membrane structure and fluidity, serve protective roles as antioxidants, and act in signal transduction (20, 21, 22). Macrophage membrane plasmalogens are essential for phagocytosis (23). In human monocyte-derived macrophages (MDMs), the glycerophospholipid (PL) mass is composed of 23% plasmenylethanolamine, 16% phosphatidylethanolamine, 4% plasmenylcholine, and 35% phosphatidylcholine (24).

Stimulation of macrophages by zymosan or Complete Freund’s adjuvant results in activation of phospholipase A_2_ (PLA_2_) enzymes that catalyze the hydrolytic cleavage of membrane diradylglycerophospholipids at *sn*-2 to release a fatty acid (often arachidonic acid) and a lysophospholipid (25, 26, 27). The hydrolytic cleavage of plasmalogen at *sn*-2 to form lysoplasmalogen and free fatty acid is shown in Figure 1, reaction 1.

**Figure 1:**
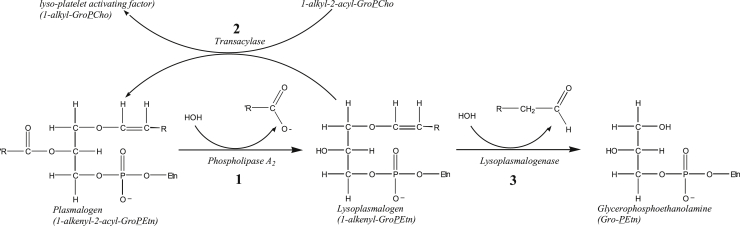
**Pathways for plasmalogen metabolism in macrophages**.

Similar to monoacylglycerophospholipids, lysoplasmalogens are amphiphiles that can insert into phospholipid bilayer membranes and alter membrane structure and function. At levels near to and above their critical micelle concentration, amphiphiles can disrupt and dissolve membrane components to cause cell injury or death (28). As such, lysoplasmalogens are normally maintained at low levels through a balance between their release by PLA_2_ and their conversion back to plasmalogens *via* a coenzyme A–independent transacylase (Fig. 1, reaction 2) (29, 30).

Alternatively, lysoplasmalogens can undergo hydrolytic cleavage at the *sn*-1 vinyl ether bond in a reaction that produces glycerophosphoethanolamine (GPE) or glycerophosphocholine (GPC) and fatty aldehyde (Fig. 1, reaction 3). This reaction is catalyzed by lysoplasmalogenase, an integral membrane protein in the endoplasmic reticulum. This enzyme activity is tissue-specific and cell type–specific and is abundant in liver parenchymal and Kupffer cells (31, 32, 33, 34), small intestinal epithelial cells (35), and certain macrophages (M.S. Jurkowitz, unpublished observations). The biological role of lysoplasmalogenase in these cell types is not known. However, they are all capable of phagocytosis, apoptosis, and/or catabolism of cell membrane or lipoprotein and phospholipid molecules.

Previously, we purified the endogenous lysoplasmalogenase enzyme from rat liver microsomes and identified the gene as *TMEM86B*, which encodes a 226-amino acid (aa) protein with eight predicted transmembrane helices (34). The protein belongs to the YhhN family, for which the function at the time was entirely unknown, despite having members in the sequenced genomes of 382 species of eukaryotes and 1638 species of bacteria, including *E. coli* (The Pfam protein families database http://pfam.xfam.org/)*.*

This work thus established a putative function for the entire family of YhhN proteins as lysoplasmalogenase enzymes. Curiously, however, many of the microorganisms that encode a YhhN family protein do not encode the machinery for synthesizing plasmalogens (16). This led us to question whether the YhhN proteins in such organisms act on lysoplasmalogens of their respective hosts or if they perhaps act on other types of molecules. To resolve this issue, we previously expressed and purified the YhhN protein from *Legionella pneumophila*, another intracellular pathogen of macrophages, and demonstrated that it indeed exhibits lysoplasmalogenase activity with very similar features to the human YhhN protein (36). While this established the likelihood that all YhhN proteins are lysoplasmalogenase enzymes, it did not address the functional role of this enzyme activity in the life cycle of *L. pneumophila*.

Here we address the potential function of the YhhN protein encoded within the *M.tb* genome. Little is known about the biochemical interactions between the membranes of *M.tb* and plasmalogens of the macrophage membrane, despite their close proximity within the phagosome. The goals of the present work were to isolate and characterize the YhhN protein from *M.tb* (MtbYhhN) and to investigate its biological role*.* To this end, we cloned and expressed the MtbYhhN protein in *M.smeg*, a fast-growing mycobacterial model, and demonstrated that it too has lysoplasmalogenase activity with properties similar to the previously characterized YhhN proteins. We further demonstrate that the choline class of lysophospholipids, both lysoplasmenylcholine (pLPC) and LPC, disrupt and lyse the membranes of *M.smeg* spheroplasts, but have no toxicity toward intact *M.smeg* cells. Spheroplasts from *M.smeg* cells overexpressing the MtbYhhN protein avoid the lytic effects of pLPC by depleting it from the medium. By contrast, overexpression of MtbYhhN does not confer protection against the toxic effect of LPC. Interestingly, the ethanolamine class of these lysophospholipids, LPE and lysoplasmenylethanolamine (pLPE), did not lyse spheroplast membranes. These results suggest that the MtbYhhN protein may function to protect mycobacterial spheroplasts against the toxic effects of host pLPC by cleaving it into nontoxic, energy-producing products. We further show that overexpression of MtbYhhN in *M.smeg* cells confers a growth advantage in human macrophages. The widespread presence of the YhhN gene across the bacterial world suggests that the lysoplasmalogenase activity of the YhhN protein may play an important role in the war between microbes and hosts.

## Results

### Cloning, expression, and purification of MtbYhhN proteins in *M.smeg*

The YhhN protein of *M.tb* is encoded by the Rv1401 gene, which could potentially express a protein of 261 amino acids. However, predicted proteins of 200, 231, 237, and 261 amino acids have appeared in different databases, depending on the gene transcription and translation start sites (all proteins end at the same stop codon). We cloned gene fragments encoding all four of these possible proteins into a mycobacterial expression vector, transformed the resulting plasmids into *M.smeg* cells, and monitored expression of the proteins after culturing the transformants in growth medium.

Harvested cells were sonicated, and whole-cell lysates were solubilized with 1% dodecylmaltoside (DDM) and assayed for total protein amount by Bradford assay (37) and for lysoplasmalogenase activity by a coupled enzyme spectrophotometric assay (33, 38). While the lysates from cells expressing the MtbYhhN proteins of 231, 237, and 261 amino acids exhibited abundant and roughly equal levels of lysoplasmalogenase activity, the 200-aa protein and the empty vector control (VC) gave about 20-fold lower levels of activity (Table 1). The presence of DDM was required for activity. These results demonstrate that the MtbYhhN protein does indeed function as a lysoplasmalogenase enzyme and that constructs as short as the 231-aa protein (residues 30–261 of the full-length protein) are fully active. It is important to note that *M.smeg* harbors its own YhhN protein (accession number: AFP39448.1), which could have accounted for the low levels of activity seen for the empty VC cells. However, the fact that overexpression of MtbYhhN led to 20-fold higher activity than the VC indicates that 95% of the activity is due to the overexpressed protein.

**Table 1:** Protein amounts and units of lysoplasmalogenase activity in sonicated *M.smeg* cells, membranes, and cytosol

Purification step	Length of MtbYhhN protein (amino acids)	Protein mg/fraction	Units nmol/min/fraction	Specific activity nmol/min/mg	Purification fold	Recovery%
Sonicated cells (75 ml)	200	83,3	198	2.4	–	100
	231	88.5	4026	45.5		
	237	47.5	2792	58.8		
	261	89.9	4780	53.2		
	Vector	95.2	224	2.3		
Solubilized membranes (5–8 ml)	200	23.9	283	11.9	5	
	231	19.6	5752	292.9	6.4	143
	237	13.7	3989	333.3	5.5	143
	261	20.1	6828	339.0	6.4	143
	Vector	24.1	320	13.3	5.8	
Cytosol (75 ml)	200	59.5	0	0		
	231	68.9		0		
	237	33.8	0	0		
	261	69.8	0	0		
	Vector	71.1	0	0		

Membrane fractions from the cells expressing the 231-, 237-, and 261-aa proteins exhibited enriched levels of specific activity, approximately 5-fold higher than for the corresponding whole-cell lysates. The levels of activity seen for the 200-aa membranes and the VC membranes were each only 4% of the levels of the overexpressing 231-, 237-, and 261-aa membranes. No activity was observed for the cytosolic portion of any of the cell lysates. A summary of these experiments is presented in Table 1.

**Figure 2:**
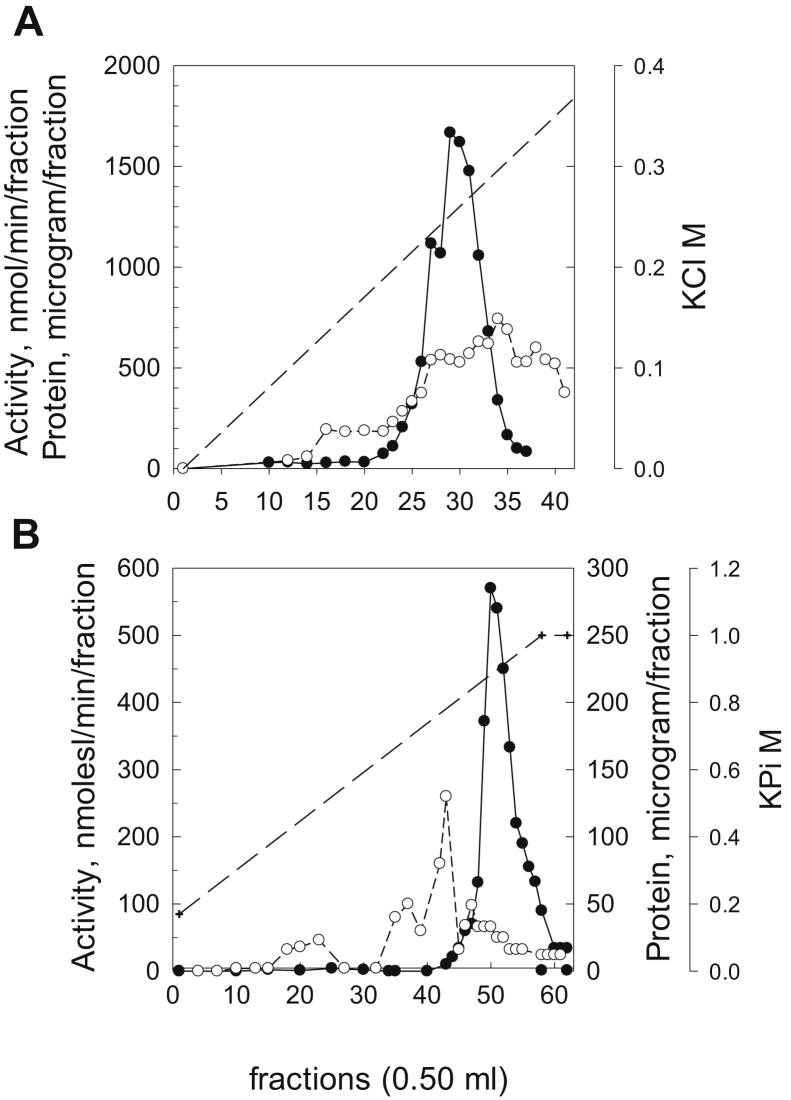
**Purification of the MtbYhhN protein of 261 amino acids.***A*, Elution profile from a Mono Q anion exchange column. *B*, Elution profile from a hydroxyapatite (CHT2) column. Lysoplasmalogenase activities (*filled circles*) were determined by the coupled enzyme spectrophotometric assay and expressed in nmol/min/fraction. The substrate was 400 μM pLPC. Protein levels (*open circles*) were determined by Bradford assay and are given in μg/fraction. The dashed line represents the salt gradient. These elution profiles are shown for one purification that is representative of three. pLPC, lysoplasmenylcholine.

The lysoplasmalogenase activities from the solubilized membrane fractions of the cells expressing the 231-, 237-, and 261-aa proteins were further purified by Mono Q and hydroxyapatite chromatography. Elution profiles from these columns were similar for all three proteins. The results of a preparation from a 600-ml culture of *M.smeg* cells expressing the 261-aa protein are presented in Figure 2. Starting with the solubilized membranes, the Mono Q column resulted in 2.5-fold purification and 51% recovery and the hydroxyapatite column resulted in a further 6-fold purification with 12% recovery (Table 2). Overall, these steps resulted in 219-fold purification, a yield of 12% and a final specific activity of 14.2 μmoles/min/mg of total protein.

**Table 2:** Summary of the partial purification of the MtbYhhN 261-aa protein

Purification step	Volume ml	Protein μg/fraction	Units nmol/min/fraction	Specific activity nmol/min/mg	Purification fold	Recovery%
1. Sonicated cells with 1% DDM		44,487	2900	65	–	<ND>
2. solubilized membrane fraction	–	16,620	16,000	963	15.3	100
3. Mono Q fractions 27–32	4.9	3371	8131	2375	36.5	51
4. Hydroxyl apatite fractions 49–52	2.8	133	1888	14,211	218.6	11.8

Enzyme activities were measured by coupled enzyme assay, and proteins by Bradford assay.

**Figure 3:**
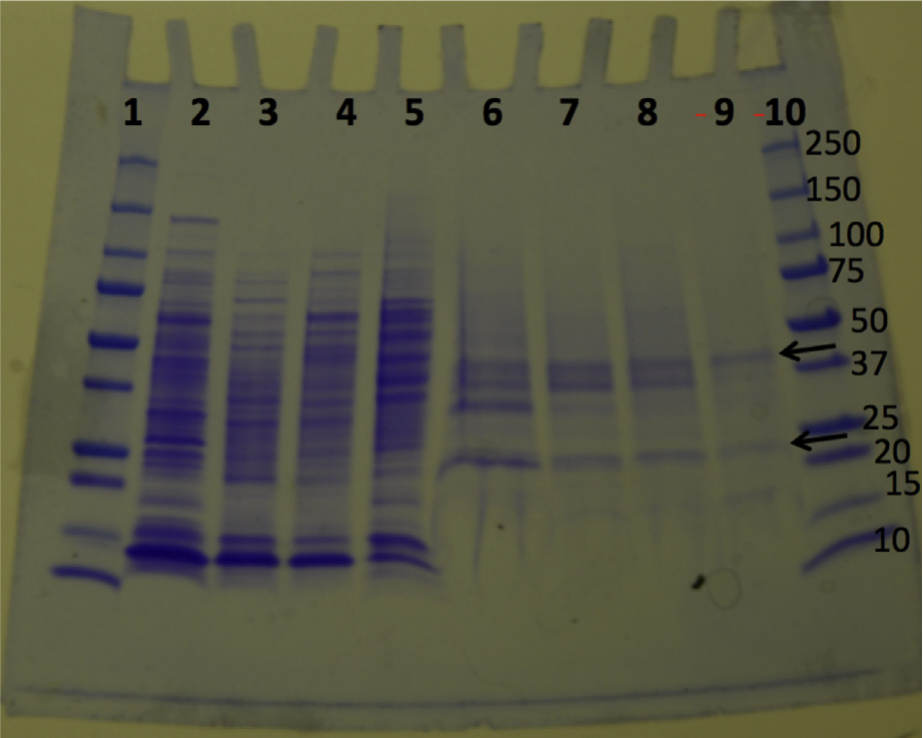
**SDS-PAGE analysis of the 261-aa MtbYhhN protein during purification.** The 4 to 20% polyacrylamide gel was stained with Coomassie. The arrows point to enriched bands at 38 and 23 kDa that were cut out of lane nine and submitted for proteomics analysis. Lanes 1 and 10, protein ladder; Lane 2, solubilized membrane fraction that was loaded onto Mono Q (19.3 units); Lane 3, Mono Q fraction 26 (9.6 units); Lane 4, Mono Q fractions 27 to 32 (32.8 units); Lane 5, Mono Q fraction 34 (14 units); Lane 6, hydroxyl apatite (HA) fractions 47 to 48 (28.7 units); Lane 7, HA fractions 49, 50 (163.8 units); Lane 8, HA fractions 51 to 52 (172.5 units); Lane 9, HA fractions 54 to 58 (50.7 units).

SDS-PAGE (39) of fractions from each step of the purification of the MtbYhhN protein of 261 amino acids is shown in Figure 3. The fractions from the final hydroxyapatite column show enrichment of two bands at 23 and 38 kDa (Fig. 3, lanes 6–9) that are close to (but lower than) the expected sizes for the monomer and dimer forms of MtbYhhN (M_r_ 27.373 kDa). The YhhN proteins of *L. pneumophila* (Lpg1991) and mammalian liver (TMEM86B) also ran lower than the actual molecular weights (34, 36). These bands were cut out of the gel and analyzed by mass spectrometry proteomic analyses at W. M. Keck MS and Proteomics Resource (Yale University School of Medicine). These studies confirmed the presence of MtbYhhN protein (P9WG51.2) in both bands. The lower band analysis is seen in Fig. S1*A*, and the higher band in Fig. S1*B* (Supporting information).

### Cloning and expression of the 200-aa MtbYhhN in *E. coli* and partial purification

The gene encoding the MtbYhhN protein of 200 amino acids gave no activity when expressed in *M.smeg* (Table 1) and thus could not be purified by tracking activity. We thus cloned and expressed it in *E. coli* as a C-terminal-GFP-His8-fusion protein. Membranes were solubilized, and the fusion protein was purified by nickel-affinity chromatography and cleaved with tobacco etch virus (TEV) protease. SDS-PAGE revealed a prominent fluorescent band at ∼36 kDa for the intact fusion protein, but after TEV treatment, two new bands appeared—a fluorescent band at 23 kDa for GFP and a nonfluorescent band at 18 kDa for MtbYhhN of 200 amino acids (Fig. S2, lanes six and 7). No lysoplasmalogenase activity was observed for either the fusion protein or the TEV cleaved protein (data not shown). These results further confirm the lack of activity from the 200-aa MtbYhhN protein.

### Kinetic and biochemical properties of the MtbYhhN proteins expressed in *M.smeg*

**Figure 4:**
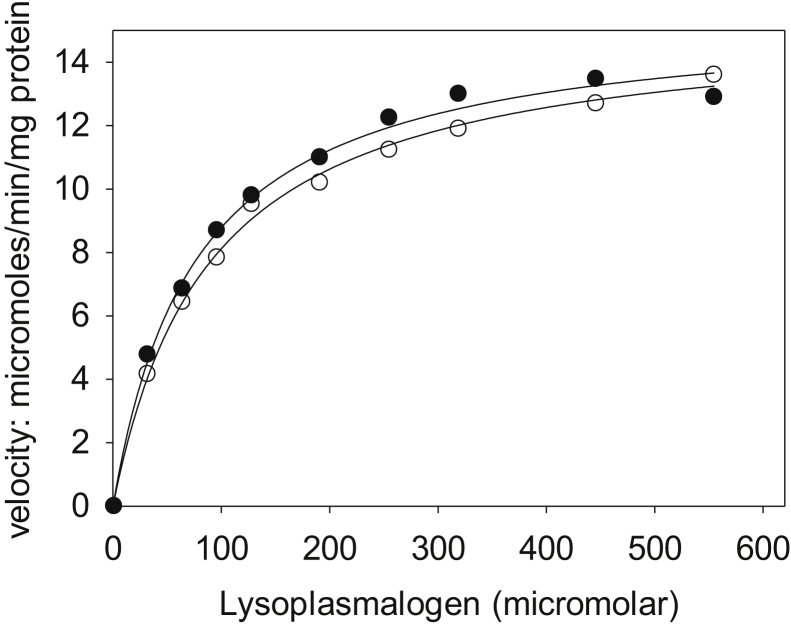
**Lysoplasmalogenase activity as a function of substrate concentration.** Reaction velocities were determined by coupled enzyme spectrophotometric assay. Substrates were pLPC (*filled circles*) or pLPE (*open circles*). One microgram of enzyme from the hydroxyl apatite column–pooled fractions 49 to 52 was added per 0.52 ml of reaction mixture. Each point represents the average of two duplicate assays, and the standard deviations were less than 20%. The experiment was typical of three experiments, using pooled fraction from a hydroxyapatite column similar to fractions 49 to 52, but from a different enzyme purification preparation. pLPC, lysoplasmenylcholine; pLPE, lysoplasmenylethanolamine.

**Figure 5:**
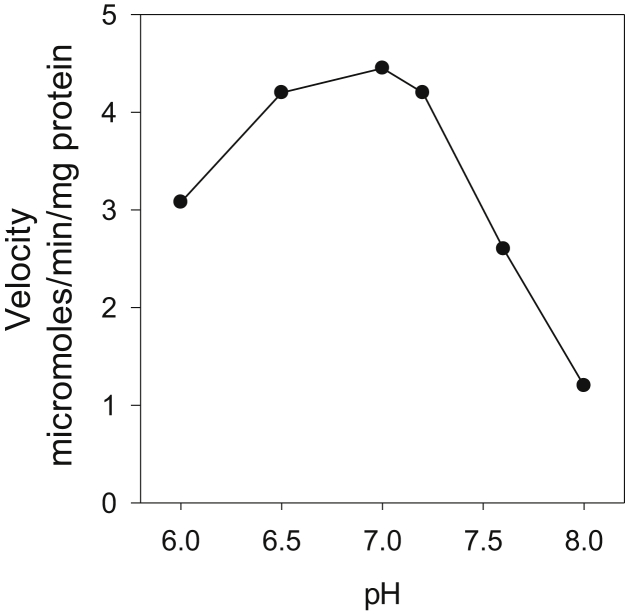
**Effect of varying pH on the hydrolysis of lysoplasmalogen (pLPC) by partially purified *M.tb* lysoplasmalogenase.** The coupled enzyme assay was used with 80 mM 3-(N-morpholino)ethansulfonic acid (NaOH) for pH 5.5 to 6.7 and 80 mM glycylglycine (NaOH) for pH 7.5 to 7.9. Two micrograms of a partially purified fraction from the hydroxyapatite column were added per 0.5 ml of incubation reaction mixture. The concentration of pLPC was 300 μM. Data are shown from one experiment representative of three experiments. pLPC, lysoplasmenylcholine.

**Figure 6:**
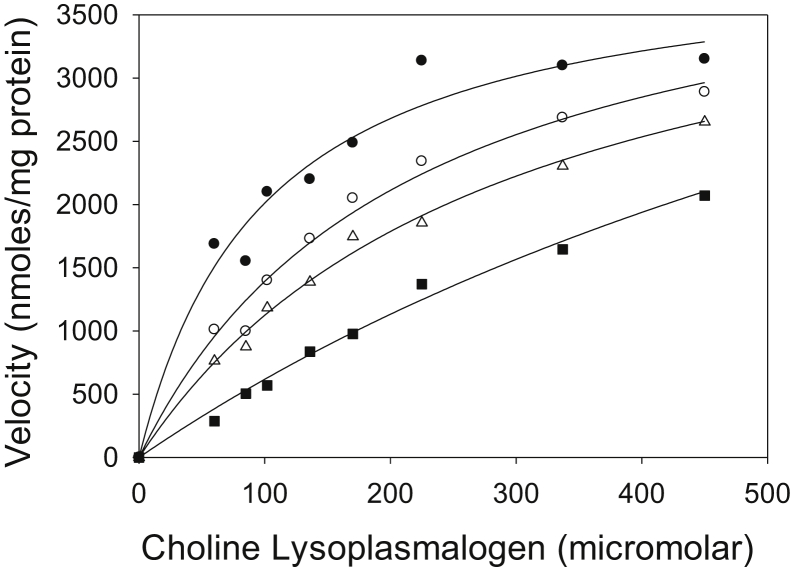
**Competitive inhibition of *M.tb* lysoplasmalogenase by lysophosphatidic acid (LPA).** Lysoplasmalogenase activity as a function of lysoplasmalogen concentration in the presence of different concentrations of LPA at pH 7.0: 0 μM (*filled circles*), 47 μM (*open circles*), 94 μM (*open triangles*), and 185 μM (*filled squares*). The enzyme activity was measured using the coupled enzyme assay. The enzyme source was 1.5 μg protein of a pooled fraction from a hydroxyapatite column similar to fractions 49 to 52, but from a different enzyme purification preparation. Data are shown from one experiment that is representative of three.

A coupled enzyme assay that detects the release of fatty aldehyde (Enzyme assay 1) was used for enzyme kinetic analysis (Figure 4, Figure 5, Figure 6). The kinetic properties of the partially purified MtbYhhN proteins of 231, 237, and 261 amino acids are very similar to one another and are reported here for the 261-aa protein. The enzyme reaction was dependent on time for >20 min and on protein concentration (data not shown). The activity of the solubilized enzyme was completely abolished by boiling for 5 min or freezing at −20 ^°^C. The rates of hydrolysis of both pLPC and pLPE were the same in the presence of N_2_ or O_2_ (air), indicating that the enzyme does not require molecular oxygen (data not shown).

The GPE product of the reaction was identified and quantified following chloroform/methanol extraction and TLC analyses of the reaction incubation, as described in Experimental procedures (Enzyme assay 2) (33). The aldehyde was quantified in parallel experiments by Enzyme assay 1. The results showed a 1:1:1 stoichiometry for the amounts of lysoplasmalogen consumed and GPE and aldehyde produced (Table S1).

The dependence of initial velocity on increasing substrate concentration was similar for pLPC and pLPE substrates (Fig. 4). The velocities followed rectangular hyperbolas’ characteristic of saturation kinetics. A fit of the data to the Michaelis–Menten equation gave apparent V_max_ values of 15.5 μmol/min/mg for both substrates and K_m_ values of 76.9 μM and 89.4 μM for pLPC and pLPE, respectively.

The activity of MtbYhhN in cleaving the vinyl ether linkage at *sn-1* was dependent on the substrate having an –OH group at sn-2: no activity was observed with plasmalogens, including 1-alkenyl-2-acylglycerophospho-ethanolamine or glycerophospho-choline substrates. The enzyme specifically hydrolyzes the vinyl ether bonds of pLPC and pLPE and has no activity toward the ester linkage of LPC or LPE, as determined by a lipid extraction and TLC assay (Enzyme assay 2).

The optimal pH for the reaction is in the range of 6.6 to 7.2. At pH 6, the velocity is 70% maximum (Fig. 5). The velocity declines rapidly at higher pH. The high activity of the enzyme under mildly acidic condition supports a role for the enzyme in protecting *M.tb* spheroplasts within the phagosome.

Lysophosphatidic acid (LPA) competitively inhibits the reaction with a *K*_*i*_ of 90 to 100 μM (Fig. 6).

These properties of the MtbYhhN enzyme are very similar to those of the previously purified YhhN proteins from human and *L. pneumophila*, indicating that the enzymatic profile for the MtbYhhN protein family is highly conserved.

### Possible biological functions of MtbYhhN

We have demonstrated that MtbYhhN is a lysoplasmalogenase that converts lysoplasmalogens to fatty aldehyde and glycerophospho-choline or glycerophospho-ethanolamine. In the context of *M.tb*, possible biological functions of MtbYhhN include a protective role in removal of toxic host lysoplasmalogen and/or a catabolic role in generating products that can be used for energy *via* glycolysis (GPE) and β-oxidation (fatty aldehyde). The following experiments address the potential protective role.

### Effects of lysophospholipids on *M.smeg* cells

**Figure 7:**
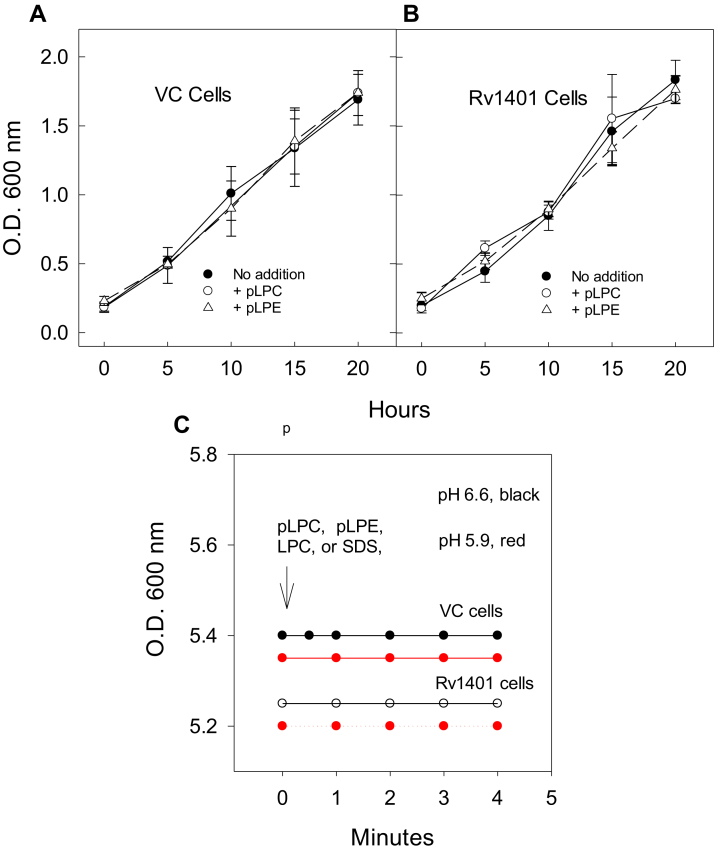
**The effects of lysoplasmenylphospholipids on growth of mycobacterial cells and the effects of lysoplasmenyl- and lysophosphatidyl-phospholipids and SDS on suspensions of mycobacterial cells.** The effects of pLPC and pLPE on the growth of *M. smegmatis* mc2155/pSMT3 (vector control: VC) cells (*A*) and mc2155/pSMT3Rv1401(1–261) [Rv1401] cells (*B*). The cells were growing in 7H9 media containing 0.5% glycerol and 11 mM glucose at pH 6.6, with and without 200 μM pLPC or pLPE. Overnight cultures of *M.smeg* cells were inoculated into the media to give an initial OD_600_ between 0.12 and 0.16. The means and S.D.s of eight experiments are shown in A and B. The cells were incubated at 170 rpm and 37 ^°^C. Aliquots were removed at indicated times for reading on a spectrophotometer. The standard error bars are shown, and the number of experiments was 7 to 9. *C*, vector control and Rv1401-overexpressing cells are incubated in 7H9 media at an initial OD_600_ of 0.53 to 0.54. The 7H9 media was either pH 6.6 or pH 5.9. The latter pH was adjusted by addition of 20 mM MES (morpholino-sulfonic acid). The cells were treated with 200 μM pLPC, pLPE, or LPC, or 0.25% sodium dodecylsulfate (SDS), at the arrow. These experiments are representative of 3 to 5 experiments. Only one line is shown, indicating there was no effect on OD_600_ by any of these compounds—the cells were resistant to lysis and at both pH 6.6 and pH 5.9. pLPC, lysoplasmenylcholine; pLPE, lysoplasmenylethanolamine.

The effects of pLPC and pLPE on growth of *M. smegmatis* cells were assessed by measuring the absorbance (OD_600_) of *M.smeg* cells growing in 7H9 liquid culture media at pH 6.6. Measurements of suspended cells at OD_600_ nm have previously been demonstrated to be proportional to bacterial colony-forming units or CFUs (40). Wildtype *M.smeg* cells (mc2155), and cells harboring empty vector (mc2155/pSMT3) (VC) or vector overexpressing the 261-aa MtbYhhN protein (mc2155/pSMT3Rv1401(1–261), were not affected by the presence of pLPC or pLPE in the culture medium. Various concentrations of pLPC and pLPE from 100 to 500 μM were assessed, and the levels were 200 μM in experiments shown in Figure 7, *A* and *B*. The cells grew at slightly accelerating rates reaching OD_600_ of about 1.7 at 20 h.

In other experiments, pLPC, pLPE, and LPC and 0.25% SDS were individually added to VC and to Rv1401 overexpressing cells incubating in 7H9 liquid media at both pH 6.6 and at pH 5.9. The OD_600_ values of these cellular suspensions were between 0.52 and 0.54 nm. There were no changes in the OD_600_ values of these cells with any of the compounds, indicating no lysis of intact cells (Fig. 7*C*). The low pH data are shown in red (Fig. 7*C*). The mycobacterial cells are resistant to lysis by the lysolipids.

When pLPC or pLPE was added to a suspension of Rv1401-overexpressing *M.smeg* cells, the lysolipids were not hydrolyzed to end products. Depletion of pLPC and pLPE required incubating mc2155/pSMT3Rv1401(1–261) cells with lysozyme, sonicating, and solubilizing the cell lysate with DDM. Based on these results, we hypothesize that pLPC and pLPE are not able to cross the cell wall of intact *M.smeg* cells to gain access to the cytoplasmic membrane. This led us to test whether LPC, pLPC, LPE, and pLPE are toxic to spheroplasts, the cell wall–deficient forms, of *M.smeg* cells. Spheroplasts have an intact cytoplasmic membrane (12).

### Formation of spheroplasts from *M.smeg* cells

We followed the classical procedures of Udou *et al*. (11, 12) with a modification that was essential for obtaining a preparation with >98% spheroplast yield and less than 2 to 3% contaminating intact cells. Following the removal of cells and debris by low-speed centrifugation, the supernatant was strained through 40-μm nylon and the filtrate was further strained through 10-μm nylon mesh material. The pure spheroplast preparation was essential for the studies involving toxicity of pLPC and LPC and also the reversion studies. When a significant proportion of the spheroplasts are cells, the OD_600_ response to pLPC or SDS is blunted because cells are not lysed by these lysolipids or the detergent. Moreover, in the reversion of spheroplast to rod-shaped cell study, it is necessary to begin with 98% spheroplasts so that increases in OD_600_ that occur are the results of newly formed cells and their division, rather than from fast division of contaminating intact cells.

**Figure 8:**
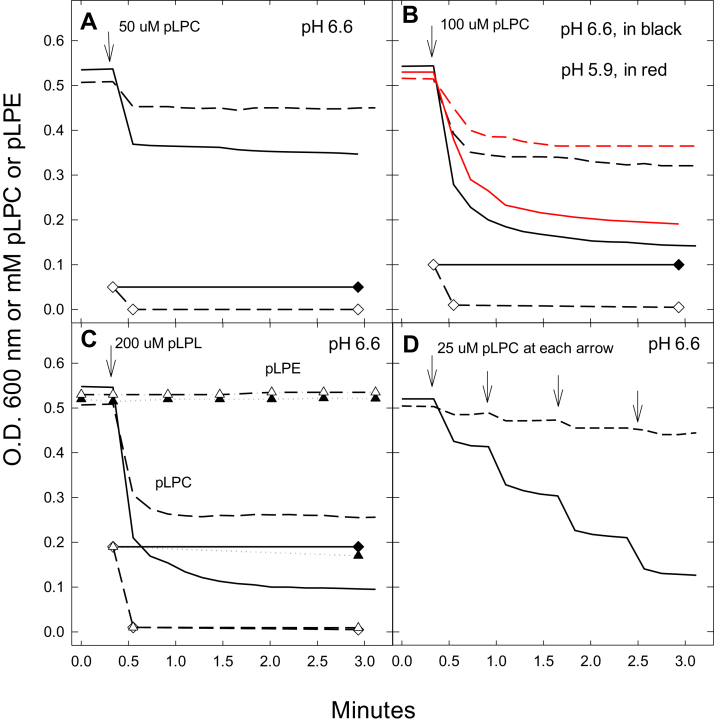
**The effects of lysoplasmenylcholine (pLPC) and lysoplasmenylethanolamine (pLPE) on OD**_**600**_**of mc2155/pSMT3 (vector control; VC) and mc2155/pSMT3Rv1401(1–261) [Rv1401] spheroplasts.** The concentrations of pLPC and pLPE (in mM) remaining in the media are shown in lower parts of graphs *A*–*C*. In A, B, and C, pLPC causes sharp decreases in OD_600_ of vector control (VC) spheroplasts (*solid lines*) that is dependent on the concentration of pLPC. The Rv1401 spheroplasts (*dashed lines*) have significantly smaller decreases in OD_600_ than the VC spheroplasts at each level of pLPC. *C*, the lack of effect of 200 μM pLPE on the OD_600_ of VC (*filled triangles*) and Rv1401 (*unfilled triangles*) spheroplasts. In (*B*), the effects of adding pLPC to VC (*red solid line*) and to Rv1401 (red dashed line) spheroplasts incubated in SMM media at pH 5.9 are shown in *red*. *A*–*C*, show the levels of pLPC (*diamonds*) or pLPE (*triangles*) remaining in the media following its addition at the arrows and rapid pulse centrifugation to remove spheroplasts from the media. These studies show the rapid depletion of pLPC from the medium in the Rv1401-expressing spheroplasts (*unfilled diamonds*). In (*C*) the level of pLPE is also rapidly depleted from the media in the Rv1401 spheroplasts (*unfilled triangles*), indicating that it is also catabolized by the MtbYhhN protein. *D*, addition of pLPC in four doses of 25 μM each to VC spheroplasts causes the same decrease in OD_600_ as when 100 μM is added as a single dose. However, when the pLPC is delivered in four doses to the Rv1401 spheroplasts, the total decrease in OD_600_ is much less than when delivered as a single bolus (*B*). These spheroplasts were suspended in SMM (500 mM sucrose, 20 mM MgCl_2_, 20 mM Na Maleate, pH 6.6 or pH 5.9). The OD_600_ measurements were determined using spectrophotometry in a Beckman DU 65 spectrophotometer. The concentrations of pLPC or pLPE remaining in the media were determined by the coupled enzyme assay. Data are shown from one experiment that is representative of seven experiments measuring OD_600_ and of three experiments measuring concentrations of pLPC and pLPE in media. pLPC, lysoplasmenylcholine; pLPE, lysoplasmenylethanolamine.

**Figure 9:**
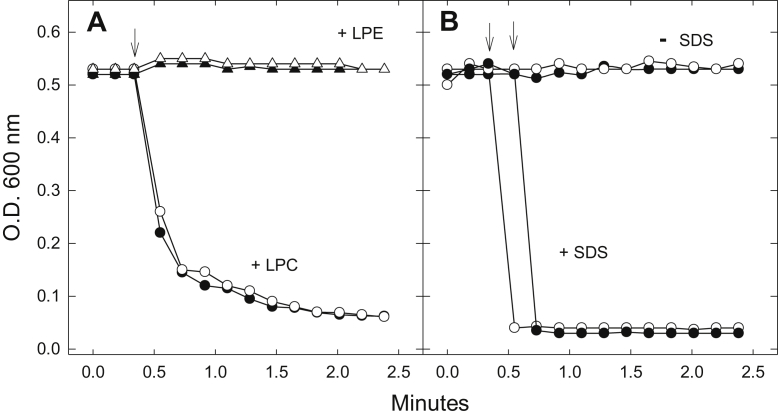
**The effects of acyl-linked lysophospholipids—lysophosphatidylcholine (LPC) and lysophosphatidylethanolamine (LPE)—and of the detergent, sodium dodecylsulfate (SDS), on the OD**_**600**_**of suspensions of spheroplasts derived from *M. smegmatis* mc2155/pSMT3 (vector control: VC) and mc2155/pSMT3Rv1401(1–261) [Rv1401] cells.***A*, 200 μM lysophosphatidylcholine (LPC) causes a sharp decline in the OD_600_ of both VC (*filled circles*) and Rv1401 spheroplasts (*unfilled circles*). 200 μM lysophosphatidylethanolamine (LPE), similar to lysoplasmenylethanolamine (pLPE), does not affect the OD_600_ of VC (*filled triangles*) or Rv1401 (*unfilled triangles*) spheroplasts. Arrow indicates the addition of LPLs. *B*, 0.25%. SDS, added at arrows, causes immediate drop in OD_600_ of both VC (*filled circles*) and Rv1401 (*unfilled circles*) spheroplasts. Data are shown from one experiment that is representative of five experiments.

**Figure 10:**
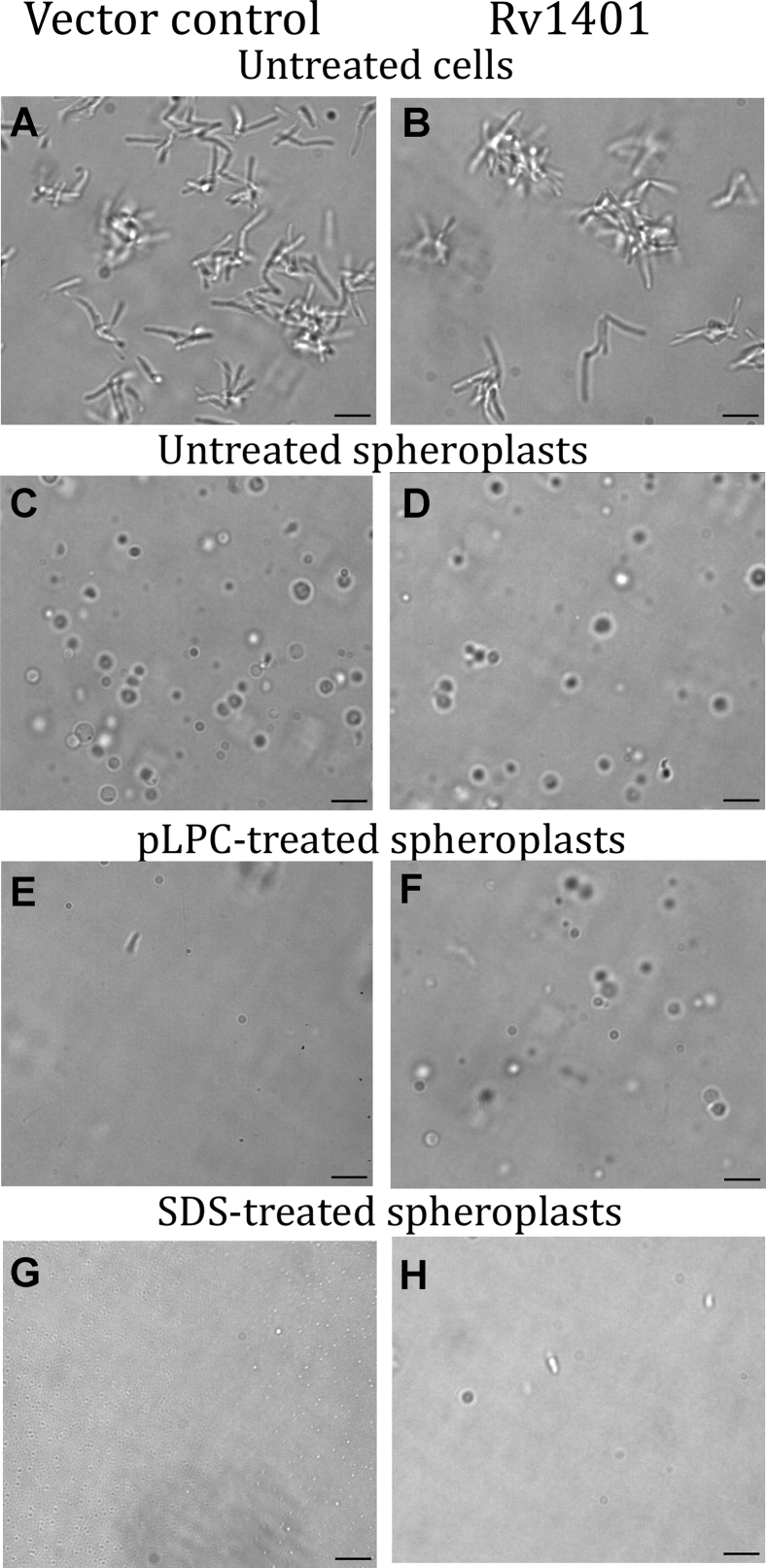
**Photomicrographs of mc2155/pSMT3 (vector control or VC) cells and spheroplasts and of mc2155/pSMT3Rv1401(1–261) [Rv1401] cells and spheroplasts.** The cells were suspended in 7H9 media supplemented with 0.5% glycerol and 11 mM glucose. The spheroplasts were suspended in SMM (500 mM sucrose, 20 mM MgCl_2_, 20 mM Na Maleate, pH 6.6). The OD_600_ measurements were determined using spectrophotometry in a Beckman DU 65 spectrophotometer. *A and B*, mc2155/pSMT3 (VC) and mc2155/pSMT3Rv1401(1–261) cells, respectively: These are control cells with no treatment. *C and D*, untreated VC and Rv1401 spheroplasts, respectively. These spheroplasts were subsequently treated with the slow addition of 100 μM pLPC (Fig. 8*D*). *E*, microscopy of the VC spheroplasts within 10 min following treatment with pLPC; note the lack of spheroplasts. *F*, the Rv1401 spheroplasts 10 min following treatment with pLPC, with many spheroplasts, similar to the untreated Rv1401 spheroplasts (*D*). *G and H,* control VC and Rv1401 spheroplasts, respectively, that have been treated with 0.25% SDS. Both panels show relatively bare fields, with tiny particles that are possibly lipid micelles. The aliquots for microscopy were taken from the experiment shown in (D). The bar is 5 μm. Magnification was 100x with oil immersion.

**Figure 11:**
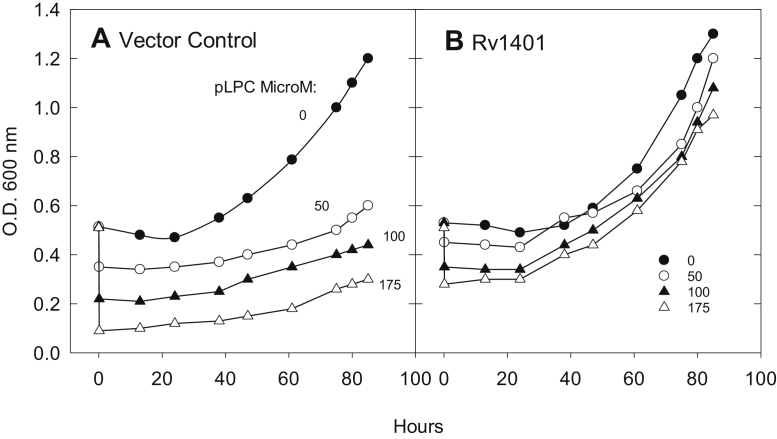
**Comparison of reversion and growth in mc2155/pSMT3 (vector control) and in mc2155/pSMT3Rv1401(1–261) [Rv1401] spheroplasts following treatment with pLPC.** The vector control and Rv1401 spheroplasts were incubated in 7H9 media with 300 mM sucrose (to maintain isotonicity), 0.5% glycerol, and 1% glucose. The initial OD_600_ of spheroplast suspensions was around 0.5 nm. The spheroplasts were then treated with pLPC; μM: 0 (*filled circles*), 50 pLPC (*unfilled circles*), 100 (*filled triangles*), and 175 (*unfilled triangles*). The 5 min OD_600_ values were recorded near the 0 time points. Subsequently, 0.52-ml aliquots were transferred to cuvettes at indicated time points over 83 h, and the OD_600_ was measured. *A*, empty vector control spheroplasts. *B*, Rv1401 spheroplasts overexpressing MtbYhhN protein. The OD_600_ of the treated Rv1401spheroplasts are increasing at faster rates than the VC-treated spheroplasts. Data are shown from one experiment that is representative of three experiments.

Procedures for quantifying spheroplasts and contaminating intact cells are given in the Experimental procedures section. In our experiments described in Figure 8, Figure 9, Figure 10, Figure 11, the preparations of spheroplasts represent ∼98% spheroplasts and ∼2% intact cells.

### pLPC is toxic to mc2155/pSMT3 (vector control) spheroplasts, and less toxic to mc2155/pSMT3Rv1401(1–261) spheroplasts overexpressing MtbYhhN

Empty VC and Rv1401-overexpressing spheroplasts were incubated in SMM media (500 mM sucrose, 20 mM MgCl_2_, 20 mM Na Maleate, pH 6.6), and OD_600_ was measured before and 5 min following addition of 50, 100, and 200 μΜ pLPC. The initial OD of spheroplasts was about 0.5 nm and remained at that level with no addition of pLPC. The addition of pLPC to VC cells led to rapid decreases in OD_600_. The major part of the decrease occurs within 30 s and may continue at a slower rate (Fig. 8, *A*–*C*, solid lines). With 50, 100, and 200 μM pLPC added to VC spheroplasts, the OD_600_ values after 3 min were 0.35, 0.14, and 0.09, respectively. The rates and the extents of the changes in OD_600_ are proportional to the concentrations of pLPC between 20 and 200 μM.

The Rv1401 spheroplasts overexpressing MtbYhhN respond to pLPC with smaller changes in OD_600_ than the empty VC spheroplasts (Fig. 8, *A*–*C*, dashed lines). With 50, 100, and 200 μM pLPC added, the OD_600_ values after 3 min were 0.45, 0.32, and 0.26, respectively. These results indicate that spheroplasts prepared from the Rv1401 cells are protected from the toxic lysolipid.

In Figure 8*C*, addition of 200 μM pLPE is shown to produce no significant changes in OD_600_ of either VC or Rv1401 spheroplasts. The lack of membrane disrupting effect by pLPE was such a surprising finding that in addition to the synthetic C-16 pLPE we also tested synthetic C-18 pLPE, as well as, pLPE prepared from porcine brain. All three of these pLPE preparations gave the same negative results with the OD_600_ values (data not shown).

We tested the hypothesis that a slow addition of pLPC to the Rv1401 spheroplasts would result in greater protection than when added as a single bolus because the enzyme would have more time to convert pLPC to nontoxic products. This experiment shows that four additions of 25 μM pLPC to Rv1401 spheroplasts resulted in overall OD_600_ change of −0.06 (Fig. 8*D*) compared with an OD_600_ change of −0.20 when 100 μM pLPC was added as a single rapid addition. The OD_600_ changes induced by slow or fast injection of 100 μM pLPC to VC spheroplasts were the same, −0.40.

To elucidate the mechanism by which MtbYhhN protects the Rv1401 spheroplasts, the amounts of pLPC in the medium of Rv1401 and VC spheroplasts were measured at various time points in experiments run in parallel to the spectrophotometric measurements.

In Rv1401 spheroplasts, the levels of pLPC rapidly declined within seconds following its addition to incubating spheroplasts (unfilled diamonds in 8A-C in lower part of graphs). By contrast, the pLPC levels in the incubation media of the VC spheroplasts remained high (Fig. 8, *A*–*C*, filled diamonds).

Figure 8*C* also shows that the level of pLPE goes to zero in the Rv1401 spheroplasts (unfilled triangles) but not in the VC spheroplasts (filled triangles).

In Figure 8*B* we examined the toxic effects of pLPC on VC and Rv1401 spheroplasts at pH 5.9 (red symbols and lines) and compared with those at pH 6.6. These experiments showed somewhat less toxicity in VC cells by pLPC at the lower pH. Thus, 1 min after the addition of 100 μM pLPC to VC spheroplasts, the OD_600_ values were 0.20 and 0.25 for pH 6.6 and pH 5.9, respectively. The protective effect in the Rv1401 spheroplasts is also greater at pH 5.9 than at pH 6.6. One min after addition of the pLPC to Rv1401 spheroplasts, the OD_600_ values were 0.34 and 0.39 for pH 6.6 and pH 5.9, respectively. The reasons for these differences may be due to the lability of the vinyl ether bond to acid hydrolysis at low pH

We conclude that the MtbYhhN protein protects mycobacterial spheroplasts against the toxic effects of pLPC by rapidly catabolizing it and depleting it from the media.

LPC is as toxic as pLPC to VC spheroplasts. However, Rv1401 spheroplasts are not protected from LPC.

It was important to determine if the toxicity of pLPC was specific to the choline lysophospholipid that contains a vinyl ether linkage at *sn-1*. Thus, we examined the effect of LPC on the spheroplasts and found that it is as toxic as pLPC to the spheroplasts. LPC at 200 μΜ caused a rapid drop in OD_600_ in VC spheroplasts (Fig. 9). The rate and the extent of the changes in OD_600_ were proportional to the concentrations of LPC between 20 and 200 μΜ (data not shown) and nearly identical to changes that were induced by pLPC.

LPC produced the same large decrease in OD_600_ in the Rv1401 spheroplasts (open circles, Fig. 9*A*) as in the VC spheroplasts (filled circles), indicating that Rv1401 spheroplasts were not protected from the acyl form of the lysolipid. This lack of protection in Rv1401 spheroplasts was expected because lysoplasmalogenase is specific for the vinyl ether bond at *sn-1* of pLPC and has no activity toward the ester bond at *sn-1* of LPC [(our present results and (31, 32, 33, 34)].

Similar to pLPE, LPE does not cause a change in OD_600_ in either VC or Rv1401 spheroplasts Figure 9*A* (triangles). Higher levels (400 μM) of LPE were also tested with no effect (data not shown). The small increase in OD_600_ at LPE addition is due to slight turbidity of the ethanolamine lysophospholipid solutions added to the spheroplasts.

When 200 μM pLPC, 200 μM LPC, or 0.25% SDS was added to the VC or Rv1401 spheroplast suspensions following the LPE or pLPE addition, a large decrease in OD_600_ occurred that was equal to that observed in the absence of the ethanolamine lysolipid. The OD_600_ decreased by about an absorbance value of 0.43 units (data not shown). These controls confirm that the spheroplasts responded typically to pLPC or detergent following LPE or pLPE addition, which indicates that the pLPE had not affected the spheroplasts in some unexplained way, making them resistant to solubilization by detergent or pLPC.

The rapid decreases in OD_600_ of empty VC spheroplasts upon addition of pLPC or LPC are very similar to changes induced by 0.25% SDS (Fig. 9*B*). This suggests that pLPC and LPC cause disruption and lysis of the VC spheroplast plasma membranes, a conclusion that is also supported by the photomicroscopic data (Fig. 10, *E*, *G* and H).

### Photomicroscopy of mc2155/pSMT3 (VC) and mc2155/pSMT3Rv1401(1–261) cells and their respective spheroplasts

Figure 10 includes photomicrographs of mc2155/pSMT3 (VC) and of mc2155/pSMT3Rv1401(1–261) cells in 10A and 10B, respectively. Panels 10C and 10D show VC and Rv1401 untreated spheroplasts, respectively. These spheroplasts were then treated with the slow addition of 100 μM pLPC, and panels E and F show microscopy of the VC and Rv1401 spheroplasts within 10 min following the treatment. In the VC panel E, few spheroplasts and a single rod cell are seen. In the Rv1401 panel F, many spheroplasts are seen, similar to the untreated Rv1401 spheroplasts (panel D). These results provide visual evidence that the VC spheroplasts have been disrupted/lysed and that the Rv1401 spheroplasts largely escaped this lysis. When the untreated VC and Rv1401 spheroplasts are treated with 0.25% SDS, in panels G and H, respectively, mainly clear fields with tiny particles, possibly lipid micelles, are seen. One possible spheroplast is seen in G and in H, and two rod shaped cells in H.

### Reversion of spheroplasts to rod-shaped cells

Udou et al have found that spheroplasts incubated in media with nutrients can regenerate their cell wall, revert to rod-shaped bacillary forms, and divide. This is a slow process that occurs over hours or days and is evident from an increase in OD_600_ and morphological changes from spherical form to rod shape as observed with microscopic analyses (11, 12).

The VC and Rv1401 spheroplasts were incubated in 7H9 media with 300 mM sucrose, with added 0.5% glycerol and 1% glucose. The initial OD_600_ values of spheroplast suspensions were 0.50 to 0.51. In the absence of lysophospholipids, the VC and the Rv1401 spheroplasts had similar responses; following small declines in OD_600_ over 25 h (less than 10%), their absorbance increased exponentially to attain OD_600_ values of ∼1.0 at 75 h (Fig. 11, *A* and *B*, filled circles).

Following addition of 50, 100, and 175 μM pLPC to VC cells, the 5-min OD_600_ values were 0.35, 0.22, and 0.09, respectively (Fig. 11*A*). These OD_600_ values then increased slowly over 75 to 85 h to obtain levels of 0.57, 0.44, and 0.30 at 80 h (Fig. 11*A*).

For the Rv1401 spheroplasts treated with pLPC, initial decreases in OD_600_ are proportional to the concentration of pLPC and are about 25 to 50% of the decrease in the VC spheroplasts. Following addition of 50, 100, and 175 μM pLPC, the 5-min OD_600_ values were 0.45, 0.35, and 0.28, respectively (Fig. 11*B*). These values remained relatively constant for 24 h and increased exponentially over the next 50 h of the experiment, approaching an OD_600_ value of 1.0 at 75 to 80 h (Fig. 11*B*). The rates of exponential rise of pLPC-treated Rv1401 spheroplasts were similar to those of the untreated Rv1401 spheroplasts.

In parallel experiments to Figure 11, *A* and *B*, the VC and Rv1401 spheroplasts were incubated in the absence of pLPC for 75 h to allow them to develop their cell wall. At 75 h, these spheroplasts were challenged with 200 μM pLPC. No changes in OD_600_ occurred in VC or Rv1401 spheroplasts, suggesting that the expected reversion to intact forms of mycobacterial cells had occurred.

Phase contrast microscopy supported the conclusion that the untreated VC spheroplasts, and the pLPC-treated or untreated Rv1401 spheroplasts, which were predominantly spherical at 17 h, had reverted back to being predominantly rod shaped over the course of the 75 h (data not shown). Preliminary evidence suggests that the presence of pLPC in the VC spheroplast cultures may slow the processes whereby the spheroplasts revert to rod-shaped cells; however, more experiments are needed to explore this phenomenon.

### Lysoplasmalogen-induced cell injury in human macrophages

In a number of different settings, macrophages can be exposed to agents that activate PLA_2_ to produce choline and ethanolamine lysophospholipids, including lysoplasmalogens. We were curious to know if lysoplasmalogens are toxic toward human macrophages and, if so, how it could relate to *M.tb* infection. Human macrophage toxicity was assessed by monitoring intracellular ATP levels and morphological changes following exposure to pLPC and pLPE.

Human MDMs were incubated in RPMI + Hepes media and lysophospholipid at 0, 50, 100, 200, and 400 μM for up to 3 h. We found that both pLPC and pLPE were toxic to macrophages and that pLPC was more toxic than pLPE. pLPC was toxic between 50 and 200 μM, while pLPE was toxic between 100 μM and 400 μM. With 200 μM pLPC, the ATP levels were 35% of control at 1 h and 8% of control at 3 h. With 400 μM pLPE, ATP levels were 55% of control at 1 h and 16% at 3 h. In positive control Triton-treated cells, the ATP levels were 22% and 0% at 1 h and 3 h, respectively (Fig. S3).

The morphologies of the macrophages were studied by observing cells with phase contrast microscopy at 3.5 h after treatment. Following lysoplasmalogen treatment, the cells progressed from smooth cell membranes at zero time controls to ruffled and broken membranes at the highest concentration. The morphology of the Triton-treated cells at 3.5 h showed significant damage to the cell membrane, with many bare nuclei observed (Fig. S3).

The time course of pLPC-induced ATP decrease in macrophages is slower than that of the pLPC-induced OD_600_ changes in *M.smeg* spheroplasts that occurred in seconds.

### Expression of MtbYhhN in *M.smeg* cells improves their viability in infected human macrophages

**Figure 12:**
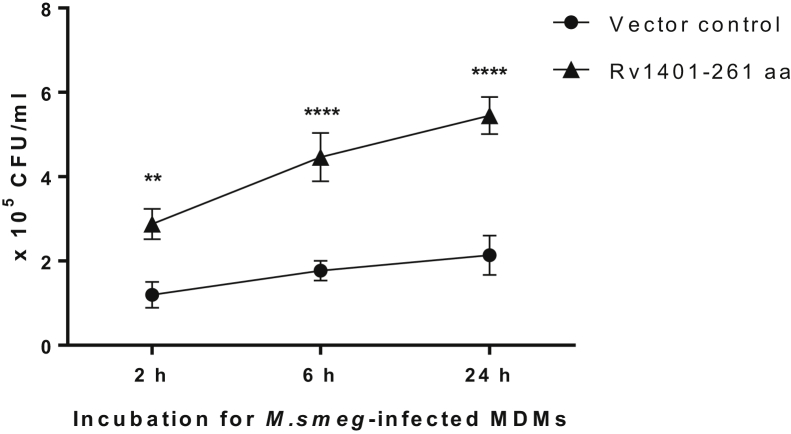
**Effects of expression of full-length active MtbYhhN protein (261 amino acids) in *M.smeg* bacterial cells on their intracellular viability in human macrophages.** Human macrophages (MDMs) were infected with either mc2155/pSMT3 (vector control; *filled circles*) or mc2155/pSMT3Rv1401(1–261) expressing MtbYhhN protein of 261 amino acids (RV1401–261 amino acids; *filled triangles*). Infected macrophages were lysed at different time points of incubation (2, 6, and 24 h) and lysates plated for bacterial growth and bacterial number counted in CFUs. Significant difference in intracellular growth was observed between *M.smeg* expressing MtbYhhN protein of 261 amino acids and *M.smeg* containing empty vector control (∗∗ *p* = 0.0035; ∗∗∗∗ *p* = <0.0001 [2-way ANOVA; Tukey’s test]). Each point is the mean of three incubations. The experiment is representative of three experiments. CFUs, colony-forming units.

We infected human MDMs with *M.smeg* transformant strains containing either empty VC or 261-aa protein–expressing construct. The Rv1401 *M.smeg* strain overexpressing the full-length active form of MtbYhhN protein (261 amino acids) showed 2.6-fold higher intracellular viability (shown in CFUs) than the empty VC (Fig. 12). Since a significant difference in CFUs was seen as early as 2 h in the MDMs, we conducted microscopy experiments to assess whether there was a difference between the *M.smeg* VC and Rv1401 strains in bacterial association with MDMs. No significant difference was observed (Fig. S4). Thus, the results confirm the activity of the 261-aa protein in a biological context and suggest that the lysoplasmalogenase (here, MtbYhhN) plays a role in mycobacterial survival in the human macrophages.

### Protein structure–function analyses

A hydropathy plot of the sequence of the 261-aa MtbYhhN protein predicts that it contains eight transmembrane (TM) alpha helices, with a C-in N-in topology (Fig. S5). “In” refers to cytoplasmic, and “out” refers to periplasmic sides of the inner membrane (41). Four proteins of different lengths are aligned with their C-terminals at amino acid 261 to convey the fact that the three smaller proteins are with N-terminal, not with C-terminal, truncations. In the full-length MtbYhhN protein, the first predicted TM helix is formed by residues 29 to 49. Since the 200-aa form of this protein consists of residues 62 to 261, it would therefore lack this helix. Assuming that all eight TM helices are necessary for the cooperative folding of the protein, this structural analysis offers a compelling explanation for why the 200-aa protein is inactive. Moreover, the second shortest protein of 231 amino acids was fully active. As it starts at residue 30, it would likely contain an intact TM1 and be correctly folded.

The 8-TM helical architecture seen in MtbYhhN is highly conserved in members of the YhhN family (42). In our previous report on the YhhN protein from *L. pneumophila*, we noted that all YhhN proteins contain five highly conserved polar amino acid residues located within the predicted TM regions (as opposed to the cytosolic loops) that could potentially form an active site (36).

**Figure 13:**
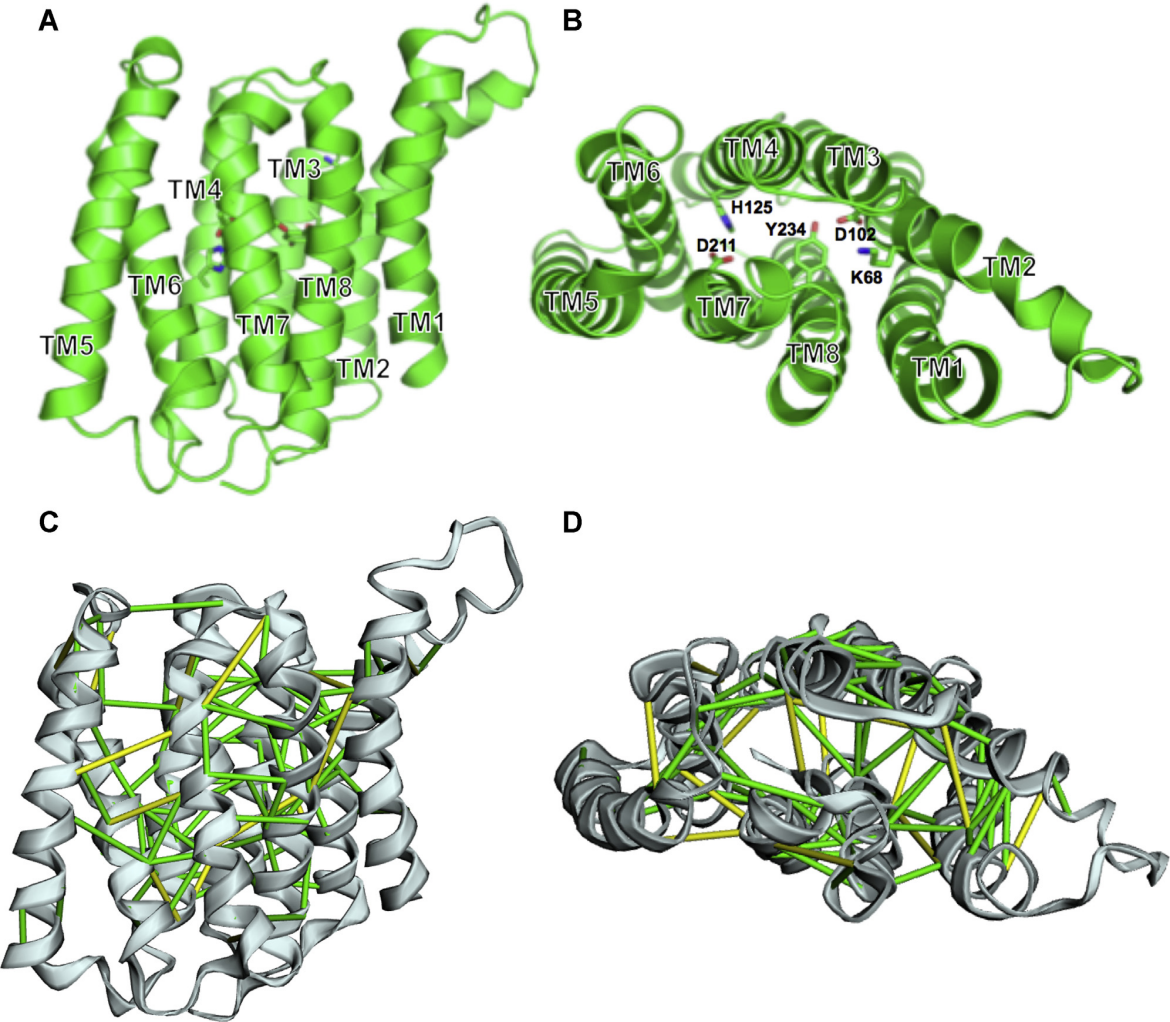
**Predicted structure of the MtbYhhN protein of 261 amino acids using covariation analysis and Rosetta energy minimization modeling programs developed by the Baker Laboratory** (42, 43). *A and C*, lateral views with periplasmic side at the top and the cytoplasmic side at the bottom. *B and D*, the structures have been rotated by 90^o^ to show the periplasmic side facing the reader. The eight transmembrane helices are numbered in *A* and *B*. In *B*, the highly conserved residues that cluster in the interior to form a putative active site (36) are shown with side chains in stick form and labeled. *C* and *D*, show coevolving residue pairs connected by green bonds for distances between coevolving residue pairs less than 5 Å and yellow bonds for distance less than 10 Å.

A model for the 261-aa MtbYhhN protein predicted by covariation analysis and Rosetta energy minimization (42, 43) is shown in Figure 13. The channel-like nature of the cavity is seen in the transverse view (Fig. 13*B*) looking down through the channel from the periplasmic side of membrane toward the cytoplasmic side. Intriguingly, the aforementioned potential active site residues (36) are clustered together within the interior of the protein and include Lys68, Asp102, His125, Asp211, and Tyr234 (Fig. 13*B*).

## Discussion

Infection with *M.tb* is initiated by inhalation of air droplets containing *M.tb* bacilli where *M.tb* surface ligands are recognized by resident macrophage receptors leading to binding and subsequent phagocytosis (5, 6, 44, 45). Most *M.tb* bacilli reside in phagosomes and are in close contact with phagosome membranes (46, 47). Within the *M.tb-*infected phagosome, the pH is mildly acidic in the range of 6.0 to 6.8 (1, 2, 3, 4). *M.tb* use host lipids for their survival (48, 49, 50, 51, 52). Relatively large amounts of the macrophage membrane glycerophospholipids are of the plasmalogen subclass (24), yet little is known about the interaction between *M.tb* and host plasmalogens or lysoplasmalogens.

### Rv1401 gene of *M.tb* encodes the MtbYhhN protein

In the present work, we found that the Rv1401 gene of *M.tb* encodes a YhhN family lysoplasmalogenase enzyme that catalyzes hydrolytic cleavage of the vinyl ether bond of lysoplasmalogens to form a fatty aldehyde and glycerophospho-ethanolamine or glycerophospho-choline (Fig. 1, Reaction 3). The function of this gene had previously been unknown. This is the first discovery of a plasmalogen-metabolizing enzyme in *M.tb*, a microbial pathogen that lacks the machinery to make plasmalogens endogenously (16).

The biochemical and kinetic properties of MtbYhhN are very similar to those of previously purified YhhN proteins from human (TMEM86A and TMEM86B) ((34), V.A. Woltz, C.E. Bell, M.S. Jurkowitz, unpublished observations) and *L. pneumophila* (Lpg1991) (36), indicating that the enzymatic profile for the YhhN protein family is highly conserved.

The vinyl ether bond cleavage activity was specific for lysoplasmalogen substrates: there was no activity on 1-alkenyl-*sn-2*-acyl-glycerophospho-ethanolamine or 1-alkenyl-*sn-2*-acyl-glycerophospho-choline (plasmalogen) substrates as the enzyme requires an –OH group at *sn-2*. Lands postulated that the hydroxyl, fixed in space near to the vinyl ether bond, may facilitate the hydrolysis (31). The catalytic mechanism is not yet known but may be related to the mechanism of hydrolysis of the vinyl ether bond of isochorismate, catalyzed by PhzD, an isochorismatase from the phenazine pathway in *Pseudomonas aeruginosa* (53). The catalytic mechanism of MtbYhhN is distinct from the recently discovered mammalian cytochrome C plasmalogenase that catalyzes oxidative and hydrolytic cleavage of the vinyl ether bond of plasmalogen to form *sn-2* acyl lysophospholipid and an alpha-hydroxy fatty aldehyde (54). This enzyme is highly selective for diradyl plasmalogen and has no activity with lysoplasmalogen (54).

### Study of the biological role(s) of MtbYhhN in *M.tb* using *M.smeg* as a model

We hypothesized that MtbYhhN may protect the cytoplasmic membrane of mycobacteria by clearance of potentially toxic lysoplasmalogens, amphiphiles that can insert into phospholipid bilayer membranes, alter membrane dynamics/function, and at higher levels dissolve membrane components (28, 55). The two products of the reaction, fatty aldehyde and GPC or GPE, contain high-energy C-H bonds that can potentially be utilized in oxidative pathways, producing ATP and NADH*.*

### Possible levels of lysolipids in macrophages during infection with *M.tb*

Although the concentrations of lysoplasmenyl and lysophosphatidyl lipids have not been measured directly, evidence suggests that the concentrations of pLPC, LPC, pLPE, and LPE within macrophages and phagosomes may reach high levels during *M.tb* infection (25, 46, 52, 56). Macrophage PLA_2_ is activated during *M.tb* infection (52, 56). Also, alveolar macrophages harvested following intravenous injection of complete Freund’s adjuvant (that contains dead *M.tb* bacilli) exhibited increases in macrophage PLA_2_ activity, leading to the release of unsaturated fatty acids, and concomitant losses in cellular phospholipids (25). Using data from the study by Freeman and Lynn (25), and from his own research (46), Kanetsuna calculated that during macrophage stimulation, the levels of free fatty acids could reach 10 mM or higher in the phagosome due to the small area of peribacillary space (see Table 2 in the study by Kanetsuna (46)). Thus by inference, the lysophospholipid levels in phagosomes may also rise to >10 mM, suggesting that the levels of pLPC and LPC used in our studies could be found *in vivo* and that our studies are relevant to pathophysiology of disease states.

### *M.smeg* cells are not lysed by lysolipids

Similar to our results with mycobacterial cells, gram-negative bacteria are resistant to the membrane perturbing effects of LPC, while gram-positive bacteria are killed by lysophospholipids (57). The resistance to membrane disruption in mycobacteria and gram-negative bacteria may be due to the relative impermeability of their thick cell wall including the PG layer that prevents the lysophospholipids from passing through and contacting the susceptible inner plasma bilayer membrane (9, 28, 57). The intrinsic resistance of gram-negative bacteria to lysophospholipids may also be due to the presence of novel resistance mechanisms mediated by a phospholipid repair system comprised of the lysophospholipid transporter and the acyltransferase in the cytoplasmic membrane (58).

### Lysoplasmenylcholine is toxic to *M.smeg* spheroplasts and overexpression of Rv1401 gene product, MtbYhhN, protects against the lysolipid

Spheroplasts, the cell wall defective forms of *M.tb*, are formed *in vitro* by glycine and lysozyme treatment, as in our studies (11, 12, 59). They also occur naturally *in vivo* in response to various stresses-antimicrobials, hormones, immune factors including lysozyme released by macrophages (60). Spheroplasts have been isolated from clinical specimens of patients affected by pulmonary TB and other diseases (61, 62). The ability of spheroplasts to resist phagocytosis to a greater extent than the bacillary form, and to replicate slowly, led to the hypothesis that the spheroplast form may play a role in the dormancy state of *M.tb* in human lung macrophages (63, 64, 65). Thus, these spheroplast forms are relevant to the life cycle of mycobacteria and to disease (13, 14).

This is the first finding that the choline class of lysophospholipids, both pLPC and LPC, are toxic to *M.smeg* spheroplasts at relatively low levels of the compounds (20–200 μM) (Figure 8, Figure 9, Figure 10, Figure 11). The injury was observed as a rapid decrease in OD_600_ and as the disappearance of spheroplasts when viewed with bright field and phase contrast microscopy. Mardh and Taylor-Robinson reported that L-phase variants of bacteria that lack a cell wall were susceptible to killing by LPC and their parent cells were resistant (57).

This is the first report of a biological effect for a YhhN protein in the microbial world: *M.smeg* spheroplasts overexpressing *M.tb* Rv1401 protein product, MtbYhhN—are protected from the toxic effects of pLPC in both the acute dissolution of the membrane assay and in the longer time frame assay measuring the ability of the spheroplasts to return to bacillary form and continue growth and cell division. The toxicity of pLPC to VC *M.smeg* spheroplasts and the protection from toxicity in Rv1401 spheroplasts occurred in media at both pH 6.6 and pH 5.9, a range comparable to *M.tb-*infected phagosomes (1, 2, 3, 4, 66).

This protection from lysis was probably due to the rapid hydrolysis of lysoplasmenylcholine by MtbYhhN, as evident from our assessment of rapid loss of pLPC from the media and also from the survival of spheroplasts in microscopic analyses. The protection occurs when pLPC is administered rapidly and was greater when delivered in small doses providing time for the enzyme to clear the lysolipid before it contacts more phospholipid bilayer. The enzyme’s high V_max_ and low K_m_ are important in the protection, maintaining the levels of lysoplasmenylcholine below its critical micelle concentration (∼20–80 μM) that could disrupt bilayer membranes (28, 55). The kinetic parameters of MtbYhhN are similar to those of lysophospholipases found in macrophage-like cell line P388D1 (67) and in *L. pneumophila* (68) that act on lysophosphatidycholine and are thought to protect the cell membranes from lysis by LPC.

The effects of SDS on *M.smeg* spheroplasts confirm studies on *Mycobacterium aurum* spheroplasts that were rapidly lysed by 0.25% SDS while the intact *M. aurum* cells were not affected by the detergent (59).

We conclude that toxicity of the choline lysophospholipids is due to their detergent-like properties that disrupt and lyse the bilayer membrane of the spheroplasts.

### Lysoplasmenylethanolamine and lysophosphatidylethanolamine do not cause lysis of the spheroplast membranes

In contrast to the choline class of lysophopholipids, pLPE and LPE did not injure *M.smeg* spheroplasts. The reason for the difference in toxicity of the two classes of lysophospholipids in these spheroplasts is not known.

In studying hemolytic activity of pLPC and pLPE on mammalian erythrocytes (red blood cell [RBC]), Gottfried and Rapport (69) found that the length of the hydrophobic acyl chain was most important in determining lytic activity, so we kept this constant (C-16) in our studies. The type of bond at sn-1 group (ether, vinyl ether, or ester) had little effect on lysis ability. pLPE was one-third as toxic as pLPC with human RBCs but equally toxic with sheep RBCs (69). pLPE (C16) is somewhat less soluble in aqueous solution than pLPC (C16). Thus, the differences in toxicity toward erythrocytes may be explained by limited dispersion of pLPE in the aqueous medium and limited access to the membrane lipids (69). This could relate to our findings.

### Lysoplasmenylcholine and lysoplasmenylethanolamine lead to ATP decline in human macrophages

The lack of membrane disrupting effect on *M.smeg* spheroplasts by pLPE and LPE is surprising since they were nearly as effective as pLPC and LPC in their membrane perturbing effects on erythrocyte membranes (69) and on glial cell injury (F. Ny and M.S. Jurkowitz, unpublished observations).

Thus, we examined the effects of pLPC and pLPE on cultured primary human macrophages by assessing intracellular ATP levels as a function of time after addition of lysophospholipid. We found that pLPC and pLPE were both toxic to macrophages, and pLPC was more toxic than pLPE. The ATP declined over minutes and hours after addition of the lysophospholipids (Fig. S3). This time course is much slower than that of the disruption of *M.smeg* spheroplast membranes that occurred in seconds. The time course of injury by pLPC and pLPE on the macrophages is similar to that of effects of pLPC and pLPE on mammalian spinal cord glial cells, assessed by lactate dehydrogenase release from the injured cells (F. Ny, and M.S. Jurkowitz, unpublished observations). This slow rate of mammalian cell injury contrasts with the rapid response of *M.smeg* spheroplasts to pLPC—a response also observed in pLPC-treated isolated rat liver mitochondria, where pLPC caused an immediate uncoupling of respiration (increase in state 2 respiration rate) and cessation of ATP production (M.S. Jurkowitz, unpublished observations).

### Overexpression of MtbYhhN in *M.smeg* confers a survival advantage in human macrophages

*M.smeg* residing in phagosomes within macrophages have less oxygen and nutrients than those grown *in vitro* in 7H9 media with glycerol and 20% oxygen. Overexpression of MtbYhhN in *M.smeg* growing in human macrophages confers a survival advantage (Fig. 12). The mechanism for increased mycobacterial survival is not known but could relate to protection from toxic effects of lysoplasmalogen and/or enhanced energy production from oxidative metabolism of GPE and GPC and fatty aldehyde *via* glycolysis and β-oxidation, respectively. Additionally, we did not find a significant difference in MDM association of the VC and 261-aa protein–expressing strains at the 2-h time point by a microscopic enumeration of cell-associated bacteria. This result indicates that expression of the 261-aa protein (MtbYhhN) offers a survival advantage for *M.smeg* in macrophages. The underlying mechanism(s) for this observation await future investigation.

*M.smeg* encodes an endogenous YhhN protein that may be active. During our protein purification, we observed some baseline activity for the VC, which was 5% of the lysoplasmalogenase activity by cells overexpressing MtbYhhN. However, at the levels of lysoplasmalogen we tested, overexpression of MtbYhhN was required to decrease the toxicity. It may be that the levels of the endogenous enzyme are enough to protect against the levels of lysoplasmalogens that *M.smeg* (and *M.tb*) would be exposed to natural settings. Alternatively, it is also possible that the genes encoding those enzymes may be upregulated in response to lysolipid exposure. Although the MtbYhhN-encoding gene Rv1401 did not show up as one of the essential virulence genes for *M.tb* in mice during a transposon mutagenesis screen (70) and was not found to be upregulated in resting or activated mouse bone marrow macrophages (71), subsequent studies have indicated that the essentiality of a gene or its regulation is dependent on the system being studied. In this case, the studies reported in mouse macrophages are not always recapitulated in primary human cells which we used in this study providing the first report of a possible virulence role for the MtbYhhN protein. RT-PCR studies comparing mRNA expression of endogenous YhhN proteins in WT *M.smeg* compared with *M.tb* showed that the level of MtbYhhN mRNA was about 2.5 times higher than the level of *M.smeg* YhhN mRNA (data not shown). It is possible that this difference in expression of the lysoplasmalogenase gene between *M.smeg* and *M.tb* may contribute to the greater virulence of *M.tb* in the host.

### Structure–function relationships of the MtbYhhN protein

In examining structure–function relationships, our work with the four different constructs of MtbYhhN protein shows that the protein activity requires a minimum of residues 30 to 261, which constitute the eight TM helices and all five putative active site residues (36). These results show that the N-terminal residues 1 to 30 are not required for *in vitro* function and their role is unknown.

A YhhN protein has not yet been crystallized to determine the 3-dimensional (3-D) structure. However, the 3-D structure of MtbYhhN protein of 261 amino acids was predicted using coevolution-based structure modeling (Fig. 13) (42, 43). This structure of MtbYhhN is similar to the first YhhN family protein to be predicted—that of *E. coli* (accession number: NP_417925.1) by the Baker Laboratory (42).

Ovchinnikov noted that the *E. coli* YhhN protein has a G-protein-coupled receptor (GPCR) topology with an N-terminal transmembrane helical extension. He further observed that the fold of this protein is highly similar to that of rhodopsin, a GPCR protein with a bound retinal cofactor, a lipid-like molecule, which binds at the equivalent site in its protein interior (42).

It is intriguing to speculate that the proteins of the YhhN and bacteriorhodopsin families have evolved from a common ancestor, from which the overall fold has remained, with the active site diverging to bind to different lipid-like substrates.

The predicted fold of the MtbYhhN protein that holds the five conserved amino acids is similar to that of GPCR proteins of mammals. It is interesting that an important ligand for one of the GPCR proteins is LPA, which has been found in the present work to competitively inhibit the MtbYhhN protein (Fig. 6).

The lysoplasmalogen substrate for the enzyme enters from the periplasmic side. The products of the reaction, fatty aldehyde and GPE, may exit the enzyme in the cytosol where they can enter glycolysis and β-oxidation pathways, respectively, forming ATP (72, 73).

***In summary***, we have made four new observations: 1) the *M.tb* Rv1401 gene, previously of unknown function, encodes a YhhN family protein, MtbYhhN, that is a lysoplasmalogenase enzyme. 2) LPC and pLPC are toxic to *M.smeg* spheroplasts grown in liquid culture, while the ethanolamine classes of these lysophospholipids are not toxic. 3) MtbYhhN overexpression protects *M.smeg* spheroplasts against lethal pLPC by depleting it from the medium. 4) Overexpression of MtbYhhN in *M.smeg* confers a survival advantage to this *mycobacterium* in human macrophages possibly by converting toxic host lysoplasmalogen to energy-producing metabolites.

## Experimental procedures

### Chemicals and reagents

C-16 lysoplasmalogen choline (852464), C-16 lysophosphatidyl choline (855675), C-16 lysoplasmalogen ethanolamine (852470), C-16 lysophosphatidyl ethanolamine (856705), C-18 lysoplasmalogen ethanolamine (852471), C-18 lysoplasmalogen choline (852471), porcine brain lysoplasmalogen (850095), and lysophosphatidic acid were from Avanti Polar Lipids, Inc.

DDM was purchased from Inalco Biochemicals. The preformed 4 to 20% acrylamide gels, the Bradford protein assay, and the hydroxyapatite CHT2 columns were from Bio-Rad. The Mono Q (5/50 Gl, 1 ml volume) column and the FPLC system were from GE Healthcare Bio-Sciences Corp. The Amicon Ultra Centrifugal filter units (4 ml) (10,000 molecular weight cutoff) were from Millipore Corporation. OADC supplement (oleic acid, albumin, dextrose, catalase), Tris base, KCl, NaCl, Hepes, mannitol, and sucrose were of reagent grade, and the 40-μm cell strainers were from Sigma-Aldrich Chemical Co. Yeast alcohol dehydrogenase (LS001070) was from Worthington Biochemical Corporation. The 10-micron nylon mesh material was from Beijing PFM Screen Trading Co, Ltd (Alibaba.com).

Aqueous suspensions of lipid substrates were prepared ∼every week from stock solutions stored in chloroform/methanol 2/1 (vol/vol) at −20 ^°^C. Aliquots of lysophospholipids were dried under N_2_ as thin films; 70 mM glycylglycine-NaOH buffer (pH 7.1) was added to make 10 mM suspensions. Suspensions were vortexed, sonicated briefly, and maintained at 2 ^°^C (33).

The wildtype *M.smeg* strain MC^2^-155 was from ATCC. The *M.tb* genomic DNA was from the H_37_R_v_ strain of *M.tb*.

### Cloning and expression of reported isoforms of *M.tb* Rv1401 gene into *M.smeg*

Four different lengths of the *M.tb* Rv1401 gene, representing four isoforms of this protein (200, 231, 237, and 261 amino acids) were cloned into the mycobacterial expression vector pSMT3 at its *Bam*HI and *Eco*RI sites. pSMT3 contains the *Mycobacterium bovis* BCG *hsp*60 promoter and start of the *hsp*60 open reading frame (ORF) followed by a multiple cloning site, enabling the insertion of genes under the control of the *hsp*60 promoter (74). Each plasmid construct was transformed by electroporation into *M.smeg* (a fast-growing *mycobacterium*) for expression studies. The pSMT3 vector carries hygromycin-resistance marker, and therefore, antibiotic hygromycin was used in 7H11 agar media to select for *M.smeg* transformants during screening. The primers used for PCR amplifying of the four gene sequences encoding Rv1401 protein isoforms are given in Table S2. MtbYhhN protein–overexpressing *M.smeg* cells were cultured overnight in 8 ml of mycobacterial 7H9 broth media (pH 6.6) containing OADC supplement, 0.2% glycerol, and 0.05% Tween 80, at 37 ^°^C and 180 rpm. The next day the small cultures were diluted 50-fold into 400 ml of the same broth and grown for 48 h at 37 ^°^C. The bacterial cells were pelleted by centrifugation at 10,000*g* and kept stored at −20 ^°^C until lysis for protein isolation.

### Cell lysis, sonication, harvest of membrane fraction, and solubilization of membranes

The frozen cell pellets were thawed and suspended in 60x pellet volumes of lysis buffer containing 25 mM Tris Cl (pH 8.0 at 0 ^°^C) and 0.25 mM EDTA. The following components were added to the cell suspensions (final concentrations): MgCl_2_ (6 mM), DNase (10 Units per ml), lysozyme (1 mg/ml), PMSF (0.1 mM), and leupeptin (0.003 mg/ml) and then were incubated on ice for 60 min. Cells were sonicated in 30-ml batches with pulses every second, for 2 min; the power level was eight, and the cycle was 30. This was repeated 6 times, with intervals in between the cycles to maintain temperatures below 5 ^°^C. A small aliquot of the sonicated cell suspensions was tested for activity after adding DDM to a final concentration of 1%.

The sonicated suspensions were made isotonic by addition of 10x Tris buffered saline (pH 8.0 at 0^o^C) and were centrifuged for 12 min at 10,000*g* to pellet the unbroken cells and membrane aggregates. The 10,000*g* supernatants were centrifuged at 130,000*g* for 60 min, and the pellets comprised the membrane fractions. The supernatant (cytosol) was saved. All pellets and cytosol were stored at –20 ^°^C.

The pellets resulting from the 130,000*g* centrifugation were suspended in sufficient 1x Tris buffered saline to make a protein concentration between 4- and 8-mg protein per ml. DDM stock in water (20%) was added dropwise to make a final concentration of 1%. Suspensions were stirred gently for 1 h and then were centrifuged for 1 h at 130,000*g* to pellet insoluble material. Aliquots of the supernatant were tested for lysoplasmalogenase enzyme activity using the coupled enzyme assay (33, 38). Protein concentrations were determined by the Bradford assay (37). Small aliquots were diluted with an equal volume of purified glycerol and stored at −20 ^°^C for enzyme characterization and study. The major fractions of the 130,000*g* supernatants were used for further purification by chromatography.

### Purification of the MtbYhhN protein using column chromatography and analysis by PAGE

Three isoforms of MtbYhhN protein (261-, 237-, and 231-aa proteins) were purified by column chromatography. A portion (5 ml) of the solubilized membrane fractions were loaded on to a mono Q (1 ml) column previously equilibrated in Mono Q buffer A (25 mM Tris Cl, pH 8 at 2 ^°^C containing leupeptin and 0.08% DDM) at a rate of 0.13 ml/min. The column was washed with three column volumes of Mono Q buffer A, and the enzyme was eluted with a 0 to 440 mM KCl gradient in Mono Q buffer A over 25 ml. Small aliquots were saved, and protein and activities were measured. The enriched fractions were pooled.

The enriched fractions from the Mono Q column were loaded on to a 2-ml hydroxylapatite column equilibrated in 10 mM KPi pH 7.1 media containing 0.2 mM CaCl_2_, 0.05 mM leupeptin, and 0.08% DDM (HA buffer A). The column was washed with 6 ml of HA buffer A, followed by elution with a 17% to 100% HA buffer B gradient over 21 column volumes, where HA buffer B contained 1.0 M KPi pH 7.1, 0.01 mM CaCl_2_, 0.05 mM leupeptin, and 0.08% DDM. Proteins and activities were measured, and enriched fractions were pooled.

Sufficient 5X loading buffer was added to each purified sample to obtain final concentrations (1 X) of 40 mM Tris-HCl pH 6.8, 10% glycerol, 2.4% SDS, 50 mM dithiothreitol, and 0.004% bromophenol blue. Samples were maintained at 25 ^°^C for ∼30 min before loading into the well. It is important that samples are not heated above 60 ^°^C because the YhhN proteins aggregate at higher temperatures (36). The fractions were analyzed using 4 to 20% gradient gels using Tris-glycine buffer and the Laemmli procedure (39). Gels were stained with Coomassie stain for visualization of protein bands. The bands at 23 and 38 kDa were excised for amino acid sequencing.

This purification of MtbYhhN 261-aa protein described earlier in the study was repeated with 400 to 600 ml of *M.smeg*-overexpressing cells several times to harvest more purified MtbYhhN protein for biochemical studies.

### Cloning and expression of the Rv1401 gene encoding the 200-aa MtbYhhN protein in *E. coli*

The cloning and expression of the 200-aa protein–encoding gene into *M.smeg* did not show any lysoplasmalogenase activity. Thus, we opted to clone and express this isoform of the gene into *E. coli* for further characterization*.*

The Rv1401 gene encoding the 200-aa protein was PCR amplified from *M.tb* genomic DNA using Pfu Turbo DNA polymerase (Stratagene). The forward primer was 5′-gccggatcccatgttgcag cccgcgttcaag-3′, and the reverse primer was 5′-gcgcgatatcctagctatccgtaggtgctgcggc-3’ (*Nde*l and *Bam*HI restriction sites are underlined). The resulting PCR product was purified (Qiagen PCR purification kit) and digested with *Nde*l and *Bam*HI restriction enzymes (New England Biolabs). After a second PCR purification step, the digested PCR product was ligated into the pWaldo-GFPd vector, also digested with *Nde*l and *Bam*HI, using the Quick Ligation kit (New England Biolabs), and transformed into chemically competent DH5α *E. coli* cells. DNA sequencing of the resulting plasmid revealed that the gene was identical to Rv1401: 200-aa length (GenBank accession number NP_215917.1). The resulting plasmid expressed the Rv1401 protein as a C-terminal GFP-8xHis fusion with an intervening site for cleavage by TEV protease. After cleavage with TEV protease, the protein will have an extra sequence at the C-terminus.

The plasmid encoding the Rv1401-GFP fusion protein was transformed into C43 (DE3) cells and grown in a 1-L culture in 75% Luria Broth/25% Terrific broth containing 50 μg/ml kanamycin, in a 3-L baffled flask at 170 rpm and 37 ^°^C. When OD_600_ reached 0.5 AU, the temperature was lowered to 30 ^°^C and expression was induced with IPTG. Cells were shaken at 170 rpm overnight and harvested by centrifugation at 8000*g* for 10 min. Cells were washed once in 10 times the pellet volume at 0 ^°^C in Buffer A (150 mM NaCl, 15 mM Tris-HCl, 15 mM NaH_2_PO_4_, 0.74 mM DTT, pH 7.9) and stored as pellets at −80 ^°^C. Cell lysis, subcellular fractionation, and solubilization of membrane fractions were carried out exactly as described earlier in the study for Rv1401-overexpressing *M.smeg* cells.

A 1-ml nickel column (GE Healthcare) charged with 0.1 M nickel substrate was equilibrated in Buffer B, and 300 mM NaCl, 50 mM NaH_2_PO_4_, 10 mM imidazole, 0.1% DDM, pH 7.9, and 10 ml of solubilized membrane were loaded onto the column at 0.2 ml/min at 2 ^°^C. The column was washed with 15 ml of Buffer B, and the fusion protein, identified by its fluorescence, was eluted by a gradient from 10 to 300 mM imidazole in Buffer B over 20 ml at 0.14 ml/min. Fractions were assayed for lysoplasmalogenase activity and total protein. The protein solution was treated with TEV protease to release the actual Rv1401 from the fusion protein; Rv1401 was identified by its position on a Coomassie-stained 11% polyacrylamide gel.

An 11% polyacrylamide gel was loaded with samples from purification sequence. The gel was visualized with fluorescence before adding Coomassie Blue dye. The samples added to the well include DDM-treated membrane, 100 K supernatant, histidine fractions 11 and 12, and the TEV-treated sample (Fig. S2).

### Coupled enzyme assay 1

For measuring lysoplasmalogenase activity, the aldehyde that is produced by hydrolysis of lysoplasmalogen is reduced to an alcohol in a second enzymatic reaction, catalyzed by exogenously added yeast alcohol dehydrogenase (ADH). In the second reaction, NADH is oxidized in a one-to-one stoichiometry between the moles of aldehyde produced and moles of NADH oxidized. The reaction is monitored at 340 nm, and the absorbance change during 3 to 10 min is converted into nmol NADH oxidized using the extinction coefficient for NADH (6220 M^−1^ cm^−1^). The standard reaction mixture contained 70 mM glycylglycine-NaOH, pH 7.1, 1 mM DTT, 0.20 mM NADH, 1 mg (250 IU) of ADH per ml, in a total volume of 0.52 ml. Efficacy of the coupling enzyme was evaluated under different conditions by the addition of 10 to 20 nmoles of tetradecanaldehyde (aqueous suspension) to the reaction assay cuvette (33, 38).

The hydroxyapatite-purified enzyme fractions were used in the enzyme characterization studies. The LPA was neutralized to pH 7.0 with KOH before adding to incubation mixture. For determining the pH optimum, the buffers in the coupled enzyme assay included the following: for pH 5.5 to 6.7, 80 mM 3-(N-morpholino)ethanesulfonic acid (NaOH); for pH 6.5 to pH 8, 80 mM glycyl-glycine (NaOH).

#### Enzyme assay 2

This assay was used in the stoichiometric study in which changes in the amounts of lysoplasmalogen, GPE, and aldehyde were measured during enzymatic hydrolysis of lysoplasmalogen (32, 33) (Table S1). In a final volume of 3 ml, 10 μg of an HA fraction was incubated in 70 mM glycyl glycine buffer, pH 7.0, at 37 ^°^C. The reaction was started by the addition of 350 μM pLPE substrate from 10 mM aqueous solution. Reactions were incubated at 37 ^°^C with gentle shaking and were stopped at 0 and 10 min by the addition of 12 ml of chloroform–methanol (2:1, vol/vol). Reactions were mixed vigorously for 3 min and centrifuged to separate layers (75). The lower phase containing unaltered lysoplasmalogen was concentrated, separated on a Silica Gel H plate, and developed with chloroform–methanol–water (3:7:3). pLPE was identified with standards and quantified with phosphorus assay (76). The upper phase that contained GPE was dried and extracted twice with methanol, concentrated and applied to a Silica Gel H plate. The plate was developed with chloroform–methanol–water (3:7:3), GPE was visualized with ninhydrin spray (33), identified with standards, and quantified using phosphorus assay (76). Recoveries were 70 to 80%.

The fatty aldehyde product was quantified in parallel experiments using the couple Enzyme assay 1. The loss of substrate molecules was compared with the gain of reaction products (Table S1).

### Methods for quantifying concentration of bacterial cells and spheroplasts

Cells were quantified using absorbance measurements at 600 nm (OD_600_) of liquid cultures and plating cells on solid agar medium and counting grown bacteria as CFUs (40). The former counts all cells—viable and nonviable—and those in the quiescent state, while the latter measures viable cells capable of reproduction.

Spheroplasts were also quantified by measuring absorbance at 600 nm. It is important that the spheroplast preparations be very pure and not contaminated by intact cells that are resistant to the effects of pLPC (and SDS). The contribution of contaminating intact cells within a spheroplast preparation was assessed by the nephelometric method. An aliquot of spheroplasts was placed in water and another into SMM, and their absorbances at 600 nm are compared. Spheroplasts are ruptured osmotically in the water, leaving the intact cells. The cells in water were counted using a Petroff-Hausser cell counter. The spheroplasts in SMM were diluted with SMM and counted.

In some experiments, the percentage of contaminating cells was quantified by placing aliquots of spheroplasts into SMM to obtain an OD_600_ of ∼0.5. SDS (0.25%) was added to one of the two suspensions and mixed. The OD_600_ values were compared. SDS lyses the spheroplasts but not the intact cells. Cells remaining in the SDS-treated SMM were counted as described earlier and compared with the numbers of spheroplasts in SMM without SDS. In the highly purified spheroplasts preparations, the OD_600_ of the SDS or water-treated suspensions approaches zero. All the spheroplast preparations used in the present studies were ∼98% spheroplasts and 2% intact cells.

#### Methods for formation of spheroplasts

Seven milliliters of overnight cultures of *M.smeg* VC, WT, and *Rv1401* cells were inoculated into 50 ml of Tryptic Soy Broth containing 1% glucose and 0.2% Tween 80 and incubated at 37 ^°^C at 180 rpm until the cells reached OD_600_ of 0.50 to 0.55 (midexponential phase) (about 6–8 h). A 20% glycine solution was added to cultures to give a final concentration of 1.2% and incubated for 16 to 20 h at 120 rpm and 37 ^°^C. Cells were harvested at 1250*g* for 15 min, and pellets were washed with 40 ml of SMM (O.5 M sucrose, 20 mM MgCl_2_, 20 mM maleate (Na), at pH 6.6. Pellets were suspended in 50 ml of Tryptic Soy Broth in SMM and glycine 1.2%. Lysozyme was added at 50 μg/ml, and cells were incubated at 37 ^°^C and 85 rpm for 15 to 20 h for spheroplast formation. The spheroplasts were centrifuged at 300*g* for 7 min to remove aggregates. The supernatent was filtered first through 40-μm cell filters and then through nylon mesh material of 10-μm openings (8 in x eight in squares of material folded twice to fit into 40- to 60-ml glass funnels, all sterilized). The filtrates were centrifuged at 3800*g* for 15 min. The very small spheroplast pellets were suspended with gentle trituration into about 7 ml of SMM to obtain OD_600_ of about 0.5 to 0.6 for the experiments presented in Figure 8, Figure 9, Figure 10. For experiments involving regeneration of the cell wall, spheroplasts were incubated in 7H9 media (without albumin) containing 300 mM sucrose, 1% glucose, and 0.5% glycerol (11, 12).

Cells and spheroplasts were imaged using a Nikon TiE inverted microscope equipped with an Andor Neo sCMOS camera, a Prior LED light source, and a 100x/1.4NA Plan-Apochromat VC oil immersion objective. Typically we used 100× and oil immersion. The microscope and camera were controlled using MetaMorph software (Molecular Devices).

### Assays for studying the effects of plasmenyl and acyl lysophospholipids on *M.smeg* spheroplasts

The acute effects of lysophospholipids on OD_600_ of suspensions of VC and Rv1401 spheroplasts in 0.52 ml of SMM media were carried out in cuvettes and observed in a DU 65 spectrophotometer at 30 ^°^C. The initial absorbance reading at 600 nm was ∼0.50. After 1 to 2 min, the lysolipid was added and suspension was mixed by rapidly inverting the cuvette 6 times. The absorbance was monitored over a 5-min period. Lysophospholipids included pLPC, pLPE, LPC, and LPE. The maleate concentration in SMM media was maintained at 20 mM, and pH adjusted with NaOH.

In the reversion studies for the regeneration of cell wall, the VC and Rv1401-expressing spheroplasts were incubated in 6.0 or 12 ml of 7H9 media (pH 6.6) with 300 mM sucrose (to maintain isotonicity), 0.5% glycerol, and 1% glucose. Albumin was omitted because it binds lysophospholipids. The tubes had a capacity of 12 or 25 ml with lids that allow air circulation. The initial OD_600_ recordings of spheroplast suspensions were around 0.5. The spheroplasts were then treated with 0, 50, 100, and 175 μM pLPC, and the 5-min OD_600_ values were recorded (Fig. 11, *A* and *B*). The initial decreases in absorbance at 600 nm were proportional to pLPC concentrations. Cells were shaken at 75 rpm at 37 ^°^C in humidified incubators for 83 h. Aliquots were removed at various times, and absorbances were read at 600 nm.

#### Microscopy experiments

The procedures described earlier for the spectrophotometric studies described in Figure 8*D* were also used for the microcopy experiments. Thirty microliters of cell or spheroplast suspensions were transferred to 35-mm dishes with glass bottoms for viewing with brightfield microscopy, at 100× and under oil immersion.

### Assay for quantifying lysoplasmalogen in media following its addition to incubating *M.smeg* spheroplasts

Spheroplasts from VC and Rv1401 cells were suspended in 7H9 media with 300 mM sucrose, 0.5% glycerol, and 1% glucose (pH 6.6). pLPC was added at zero time. At designated times, the suspensions were pulse-centrifuged at 14000*g* for 20 s to pellet the spheroplasts. The supernatant was tested for the amount of pLPC remaining. Thirty to 60 s was the earliest time point. The method requires removing spheroplasts from media because spheroplasts have an NADH oxidase activity that interferes with the coupled enzyme assay. Aliquots of the supernatant were mixed 1 to 1 with 2× coupled enzyme assay mix. Purified lysoplasmalogenase from *L. pneumophila* was added. ADH was coupling enzyme 2, as described earlier. The rates of aldehyde formation were proportional to concentrations of lysoplasmalogen. A standard curve with known amounts of lysoplasmalogen was prepared under the same conditions.

### Mass spectrometry analyses of peptide fragments followed by protein identification

The predominant bands at ∼23 kDa and 38 kDa from a Coomassie-stained gel were excised and sent to the Keck MS Proteomics Resource at Yale University School of Medicine where they were digested and analyzed to identify peptides and their corresponding proteins. Briefly, the gel slices were first washed with 50% acetonitrile/50% water followed by 50 mM ammonium bicarbonate/50% acetonitrile/50% water. It was then dried, rehydrated with trypsin in 10 mM ammonium bicarbonate, and digested at 37 ^°^C for 16 h. The resultant peptides were separated using a Waters Symmetry C18 trap column and a nanoAcquity UPLC separating column.Elution was performed with a 52-min linear gradient of 5 to 50% acetonitrile in 0.05% formic acid. The LC MS/MS mass spectral data obtained from an LTQ-Orbitrap Elite mass spectrometer system were searched for analogy to known peptides/protein using the Mascot algorithm (version 2.20) for uninterpreted MS/MS spectra and searching the NCBInr database. The significance score relied on a probability-based protein/peptide identification score through searching known databases with a peptide score cutoff of 95% confidence in identification (Fig. S1, *A* and *B*). The mass spectrometry proteomics data have been deposited to the ProteomeXchange Consortium *via* the PRIDE (77) partner repository with the dataset identifier PXD027875.

### Colony-forming unit growth assay of MtbYhhN-overexpressing *M.smeg* in human macrophages

Peripheral blood mononuclear cells (PBMCs) were obtained from healthy human donors, and MDM cultures were generated as described (78). Briefly, heparinized blood was layered on a Ficoll-Paque cushion to allow for collection of PBMCs which were cultured in RPMI (Life Technologies) with 20% autologous serum in Teflon wells (Savillex) for 5 days at 37 °C/5% CO_2_ to allow for differentiation of monocytes into MDMs. PBMCs were harvested and MDMs adhered to 24-well tissue culture plates (1.5 × 10^6^ PBMCs/well) for 2 h in RPMI with 10% autologous serum, and then lymphocytes were washed away to achieve purified MDMs in monolayer culture (with >99% purity and yielding 1.5 × 10^5^ MDMs/well). The MDM monolayer was incubated with *M.smeg* containing either pSMT3 empty vector (VC) or a full-length functional variant mc2155/pSMT3Rv1401(1–261) at a multiplicity of infection of five for periods of 2, 6, and 24 h. At each time point, the infected macrophage monolayer was lysed and dilutions of lysates were plated on 7H11 agar media containing hygromycin (50 μg/ml) for 2 to 3 days to allow for bacterial growth, followed by bacterial counting for CFU assessment.

### Microscopy assessment of cell association of *M.smeg* strains with human macrophages

MDMs were cultured on coverslips in a 24-well culture plate (2 × 10^5^ cells/well) in duplicate for each time point and infected with either *M.smeg* mc2155/pSMT3 (VC) or the mc2155/pSMT3Rv1401(1–261) at a multiplicity of infection of 5. After 1 or 2 h of incubation, the infected macrophage monolayer was washed with Dulbecco's phosphate buffered saline, fixed with 4% paraformaldehyde, and then subjected to auramine–rhodamine staining (Beckton Dickinson). The coverslips from each time point were examined by confocal fluorescence microscopy, counting the stained red bacteria associated with each macrophage. Approximately 100 macrophages were examined for each type of *M.smeg* strain.

### Effects of lysoplasmenylcholine and lysoplasmenylethanolamine on macrophage viability assessed by ATP levels

PBMCs were obtained from healthy human donors, and MDM cultures were generated as described in the previous section and in the study by Schlesinger (45). Macrophages were grown in 96-well plates (5 × 10^4^ MDMs/well) in RMPI media containing autologous serum. On the day of experiment, cells were rinsed with prewarmed RPMI and then were repleted with RPMI + Hepes media (without any serum or dye) containing 0, 50, 100, 200, and 400 μM pLPE or pLPC; cells were incubated at 37 ^°^C for 0, 60, 120 and 180 min. At the indicated time, 100 μl of CellTiter-Glo 2.0 reagent was added to the cell culture medium present in each well. Plates were wrapped in aluminum foil, and the contents were mixed for 2 min at room temperature (RT) on an orbital shaker to induce cell lysis. Plates were incubated further at RT for 10 min, and luminescence (in RLUs) was read in a multiwell luminometer. An additional plate containing untreated control and lysolipid-treated cells was observed with a phase contrast microscope and photographed at 3.5 h post treatment.

## Data availability

All of the data described in the manuscript are located within the manuscript and/or within the Supporting Information that accompanies this manuscript.

The raw mass spectrometry data have been deposited in the public accessible repository, PRIDE, and are available *via* ProteomeXchange with identifier PXD027875. Reviewers can utilize the log access information Username: pxd027875@ebi.ac.uk and Password: pKta4iZR to access the deposited MS data prior to publication.

## Supporting information

This article contains supporting information.

## Conflict of interest

The authors declare that they have no conflicts of interest with the contents of this article

## References

[1] Rohde K., Yates R.M., Purdy G.E., Russell D.G. (2007). *Mycobacterium tuberculosis* and the environment within the phagosome. Immunol. Rev..

[2] Sturgill-Koszycki S., Schlesinger P.H., Chakraborty P., Haddix P.L., Collins H.L., Fok A.K., Allen R.D., G luck S.L., Heuser J., Russell D.G. (2004). Lack of acidification in Mycobacterium phagosomes produced by exclusion of the vesicular proton-ATPase. Nature.

[3] Vergne I., Fratti R.A., Hill P.J., Chua J., Belisle J., Deretic V. (2004). *Mycobacterium tuberculosis* Phagosome maturation arrest: Mycobacterial phosphatidylinositol analog phosphatidylinositol mannoside stimulates early endosomal fusion. Mol. Biol. Cell.

[4] Clemens D.L., Horwitz M.A. (1995). Characterization of the *Mycobacterium tuberculosis* phagosome and evidence that phagosomal maturation is inhibited. J. Exp. Med..

[5] Weiss G., Schwaible U.E. (2015). Macrophage defense mechanisms against intracellular bacteria. Immunol. Rev..

[6] Rajaram M.V.S., Ni B., Dodd C.E., Schlesinger L.S. (2014). Macrophage immunoregulatory pathways in tuberculosis. Semin. Immunol..

[7] Lovewell R.R., Sassetti C.M., VanderVen B.C. (2016). Chewing the fat: Lipid metabolism and homeostasis during M. tuberculosis infection. Curr. Opin. Microbiol..

[8] Maitra A., Munshi T., Healy J., Martin L.T., Vollmer W., Keep N.H., Bhakta S. (2019). Cell wall peptidoglycan in *Mycobacterium tuberculosis*: An achilles’ heel for the TB-causing pathogen. FEMS Microbiol. Rev..

[9] Brennan P.J., Nikaido H. (1995). The envelope of mycobacteria: The outer membrane of Mycobacteria is relatively impermeable to molecules relative to mammalian membranes. Annu. Rev. Biochem..

[10] Mattman L.H. (2001).

[11] Udou T., Ogawa M., Mizuguchi Y. (1982). Spheroplast formation of *Mycobacterium smegmatis* and morphological aspects of their reversion to the bacillary form. J. Bacteriol..

[12] Udou T., Ogawa M., Mizuguchi Y. (1983). An improved method for the preparation of mycobacterial spheroplasts and the mechanism involved in the reversion to bacillary form: Electron microscopic and physiological study. Can. J. Microbiol..

[13] Domingue G. (2010). Demystifying pleomorphic forms in persistence and expression of disease. Discov. Med..

[14] Slavchev G., Michailova L., Markova N. (2016). L-form transformation phenomenon in *Mycobacterium tuberculosis* associated with drug tolerance to ethambutol. Int. J. Mycobacteriol.

[15] Markova N., Slavchev G., Michailova L. (2015). Presence of mycobacterial L-forms in human blood: Challenge of BCG vaccination. Hum. Vaccin. Immunother..

[16] Goldfine H. (2010). The appearance, disappearance and reappearance of plasmalogens in evolution. Prog. Lipid Res..

[17] Braverman N.E., Moser A.B. (2012). Functions of plasmalogen lipids in health and disease. Biochim. Biophys. Acta.

[18] Rapport M.M., Lerner B., Alonzo N., Franzl R.E. (1957). The structures of plasmalogens. II. Crystalline lysophosphatidal ethanolamine (acetal phospholipid). J. Biol. Chem..

[19] Gross R.W. (1984). High plasmalogen and arachidonic acid content of canine myocardial sarcolemma: A fast atom bombardment mass spectroscopic and gas chromatography-mass spectroscopic characterization. Biochemistry.

[20] Ford D.A., Gross R.W. (1989). Plasmenylethanolamine is the major storage depot for arachidonic acid in rabbit vascular smooth muscle and is rapidly hydrolyzed after angiotensin II stimulation. Proc. Natl. Acad. Sci. U. S. A..

[21] Zoeller R.A., Morand O.H., Raetz C.R.H. (1988). A possible role for plasmalogens in protecting animal cells against photosensitized killing. J. Biol. Chem..

[22] Brites H.R., Waterham R.J.A., Wanders R.J. (2004). Functions and biosynthesis of plasmalogens in health and disease. Biochim. Biophys. Acta.

[23] Rubio J.M., Astudillo A.M., Casas J., Balboa M.A., Balsinde J. (2018). Regulation of phagocytosis in macrophages by membrane ethanolamine plasmalogens. Front. Immunol..

[24] Wallner S., Orso E., Grandl M., Konovalova T., Liebisch G., Schmitz G. (2018). Phosphatidylcholine and phosphatidylethanolamine plasmalogens in lipid loaded human macrophages. PLoS One.

[25] Freeman B.A., Lynn W.S. (1980). Fatty acid secretion and metabolism in ‘activated’ rabbit alveolar macrophages. Biochim. Biophys. Acta.

[26] Gil-de-Gomez L., Astudillo A.M., Lebrero P., Balboa M.A., Balsinde J. (2017). Essential role for ethanolamine plasmalogen hydrolysis in bacterial lipopolysaccharide priming of macrophages for enhanced arachidonic acid release. Front. Immunol..

[27] Gaposchkin D.P., Harrison W.F., Zoeller R.A. (2008). On the importance of plasmalogen status in stimulated arachidonic acid release in the macrophage cell line RAW 264.7. Biochim. Biophys. Acta.

[28] Weltzien H. (1979). Cytolytic and membrane-perturbing properties of lysophosphatidyl-choline. Biochim. Biophys. Acta.

[29] Kramer R.M., Deykin D. (1983). Arachidonoyl transacylase in human platelets. Coenzyme A-independent transfer of arachidonate from phosphatidylcholine to lysoplasmenylethanolamine. J. Biol. Chem..

[30] Astudillo A.M., Balgoma D., Balboa M.A., Balsinde J. (2012). Dynamics of arachidonic acid mobilization by inflammatory cells. Biochim. Biophys. Acta.

[31] Warner H.R., Lands W.E. (1961). The metabolism of plasmalogen: Enzymatic hydrolysis of the vinyl ether. J. Biol. Chem..

[32] Gunawan J., Debuch H. (1981). Liberation of free aldehyde from 1-(1-alkenyl)-*sn-*glycero-3-phosphoethanolamine (lysoplasmalogen) by rat liver microsomes. Hoppe Seylers Z. Physiol. Chem..

[33] Jurkowitz-Alexander M., Ebata H., Mills J.S., Murphy E.J., Horrocks L.A. (1989). Solubilization, purification and characterization of lysoplasmalogen alkenylhydrolase (lysoplasmalogenase) from rat liver microsomes. Biochim. Biophys. Acta.

[34] Wu L.-C., Pfeiffer D.R., Calhoon E.A., Madiai F., Marcucci G., Liu S., Jurkowitz M.S. (2011). Purification, identification, and cloning of lysoplasmalogenase, the enzyme that catalyzes hydrolysis of the vinyl ether bond of lysoplasmalogen. J. Biol. Chem..

[35] Jurkowitz M.S., Horrocks L.A., Litsky M.L. (1999). Identification and characterization of alkenyl hydrolase (lysoplasmalogenase) in microsomes and identification of a plasmalogen-active phospholipase A_2_ in cytosol of small intestinal epithelium. Biochim. Biophys. Acta.

[36] Jurkowitz M.S., Patel A., Wu L.,-C., Krautwater A., Pfeiffer D.R., Bell C.E. (2015). The YhhN protein of Legionella pneumophila is a lysoplasmalogenase. Biochim. Biophys. Acta.

[37] Bradford M.M. (1976). A rapid and sensitive method for the quantitation of microgram quantities of protein utilizing the principle of protein-dye binding. Anal. Biochem..

[38] Jurkowitz-Alexander M.S., Hirashima Y., Horrocks L.A., Dennis E.A. (1991).

[39] Laemmli U.K. (1970). Cleavage of structural proteins during the assembly of the head of bacteriophage T4. Nature.

[40] Penuelas-Urquides K., Villarreal-Travino L., Silva-Ramirez B., Rivadeneyra-Espinosa L., Said-Fernandez S., Bermudez de Lion M. (2013). Measuring of *Mycobacterial tuberculosis* growth. A correlation of the optical measurements with colony forming units. Braz. J. Microbiol..

[41] Krogh A., Larsson B., von Heijne G.I., Sonnhammer E.L. (2001). Predicting transmembrane protein topology with a hidden Markov model: Application to complete genomes. J. Mol. Biol..

[42] Ovchinnikov S., Kinch L., Park H., Liao Y., Pei J., Kim D.E., Kamisetty H., Grishin N.V., Baker D. (2015). Large-scale determination of previously unsolved protein structures using evolutionary information. Elife.

[43] Ovchinnikov S., Park H., Varghese N., Huang P.-S., Paviopoulos G.A., Kim D.E., Kamisetty H., Kyrpides N.C., Baker D. (2017). Protein determination using metagenome sequence data. Science.

[44] Guirado E., Schlesinger L.S., Kaplan G. (2013). Macrophages in tuberculosis: Friend or foe. Semin. Immunopathol..

[45] Schlesinger L.S. (1993). Macrophage phagocytosis of virulent but not attenuated strains of *Mycobacterium tuberculosis* is mediated by mannose receptors in addition to complement receptors. J. Immunol..

[46] Kanetsuna F. (1985). Bactericidal effect of fatty acids on mycobacteria with particular reference to the suggested mechanism of intracellular killing. Microbiol. Immunol..

[47] Kondo E., Yasuda T., Kanai K. (1982). Electron microscopic demonstration of close contact between intracellular mycobacteria and the phagosomal membrane. Jpn. J. Med. Sci. Biol..

[48] Barisch C., Soldati T. (2017). Breaking fat! how mycobacteria and other intracellular pathogens manipulate host lipid droplets. Biochimie.

[49] Guirado E., Schlesinger L.S. (2013). Modeling the Mycobacterium tuberculosis granuloma–the critical battlefield in host immunity and disease. Front. Immunol..

[50] Russell D.G., Cardona P.J., Kim M.J., Allain S., Altare F. (2009). Foamy macrophages and the progression of the human tuberculosis granuloma. Nat. Immunol..

[51] Daniel J., Maamar H., Deb C., Sirakova T.D., Kolattukudy P.E. (2011). Mycobacterium tuberculosis uses host triacylglycerol to accumulate lipid droplets and acquires a dormancy-like phenotype in lipid-loaded macrophages. PLoS Pathog..

[52] Duan L., Gan H., Arm J., Remold H.G. (2001). Cytosolic phospholipase A_2_ participates with TNF-alpha in the induction of apoptosis of human macrophages infected with Mycobacterium tuberculosis H37Ra. J. Immunol..

[53] Parsons J.F., Calabrese K., Eisenstein E., Ladner J.E. (2003). Structure and mechanism of *Pseudomonas aeruginosa* PhzD, an isochorismatase from the phenazine biosynthetic pathway. Biochemistry.

[54] Jenkins C.M., Yang K., Liu G., Moon S.H., Dilthey B.G., Gross R.W. (2018). Cytochrome c is an oxidative stress-activated plasmalogenase that cleaves plasmenylcholine and plasmenylethanolamine at the *sn*-1 vinyl ether linkage. J. Biol. Chem..

[55] Corr P.B., Gross R.W., Sobel B.E. (1984). Amphipathic metabolites and membrane dysfunction in ischemic myocardium. Circ. Res..

[56] Rajaram M.V., Brooks M.N., Morris J.D., Torrelles J.B., Azad A.K., Schlesinger L.S. (2010). Mycobacterium tuberculosis activates human macrophage peroxisome proliferator-activated receptor gamma linking mannose receptor recognition to regulation of immune responses. J. Immunol..

[57] Mardh P.A., Taylor-Robinson D. (1974). The susceptibility of aerobic and anaerobic bacteria, L-phase variants, candida, protozoa and viruses to lysolecithin. Acta Pathol. Microbiol. Scand. B Microbiol. Immunol..

[58] Lin Y., Bogdanov M., Lu S., Guan Z., Margolin W., Weiss J., Zheng L. (2018). The phospholipid-repair system LplT/Aas in Gram-negative bacteria protects the bacterial membrane envelope from host phospholipase A2 attack. J. Biol. Chem..

[59] Rastogi N., David H.L. (1981). Ultrastructural and chemical studies on wall-deficient forms, spheroplasts and membrane vesicles from mycobacterium aurum. J. Gen. Microbiol..

[60] Rosu V., Bandino E., Cossu A. (2013). Unraveling the transcriptional regulatory networks associated to mycobacteria l cell wall defective form induction by glycine and lysozyme treatment. Microbiol. Res..

[61] Michailova L., Kussovski V., Radoucheva T., Jordanova M., Berger W., Rinder H., Markova N. (2005). Morphological variability and cell wall deficiency in Mycobacterium tuberculosis ‘heteroresistant’ strains. Int. J. Tuberc. Lung Dis..

[62] Seiler P., Ulrichs T., Bandermann S., Pradl L., Jorg S., Krenn V., Morawietz C., Kaufmann S.H.E., Aichele P. (2003). Cell wall alterations as an attribute of Mycobacterium tuberculosis in latent infection. J. Infect. Dis..

[63] Harwick H.J., Barajas L., Montgomerie J.Z., Kalmanson G.M., Guze L.N. (1972). Phagocytosis of microbial L forms. Infect. Immun..

[64] Harwick H.J., Kalmanson G.M., Guze L.N. (1977). Chemotactic activity of L-forms and *mycoplasma*. Aust. J. Exp. Biol. Med. Sci..

[65] Onwuamaegbu M.E., Belcher R.A., Soare C. (2005). Cell wall-deficient bacteria as a cause of infections: A review of the clinical significance. J. Int. Med. Res..

[66] Queval C.J., Brosch R., Simeone R. (2017). The macrophage: A disputed fortress in the battle against Mycobacterium Tuberculosis. Front. Microbiol..

[67] Zhang Y.Y., Dennis E.A. (1988). Purification and characterization of a lysophospholipase from a macrophage-like cell line P388D1. J. Biol. Chem..

[68] Flieger A., Neumeister B., Cianciotto N.P. (2002). Characterization of the gene encoding the major secreted lysophospholipase of *Legionella pneumophila* and its role in detoxification of lysophosphophatidyl choline. Infect. Immun..

[69] Gottfried E.L., Rapport M.M. (1963). The biochemistry of plasmalogens: II. Hemolytic activity of some plasmalogen derivatives. J. Lipid Res..

[70] Zhang Y.J., Reddy M.C., Ioerger T.R., Rothchild A.C., Dartois V., Schuster B.M., Trauner A., Wallis D., Galaviz S., Huttenhower C., Sacchettini J.C., Behar S.M., Rubin E.J. (2013). Tryptophan biosynthesis protects mycobacteria from CD4 T cell mediated killing. Cell.

[71] Schnappinger D., Ehrt S., Voskuil M.I., Liu Y., Mangan J.A., Monahan M., Dolganov G., Efron B., Butcher P.D., Nathan C., Schoolnik G.K. (2003). Transcriptional adaptation of *Mycobacterium tuberculosis* within macrophages: Insights into the phagosomal environment. J. Exp. Med..

[72] Fozo E.M., Rucks E.A. (2016). The making and taking of lipids: The role of bacterial lipid synthesis and the harnessing of host lipids in bacterial pathogenesis. Adv. Microb. Physiol..

[73] Cook G.M., Hards K., Vilcheze C., Hartman T., Berney M. (2014). Energetics of Respiration and oxidative phosphorylation in mycobacteria. Microbiol. Spectr..

[74] Garbe T.R., Barathi J., Barnini S., Zhang Y., Abou-Zeid C., Tang D., Mukherjee R., Young D.B. (1994). Transformation of mycobacterial species using hygromycin resistance as selectable marker. Microbiology.

[75] Bligh E.G., Dyer W.J. (1959). A rapid method of total lipid extraction and purification. PMID: 13671378. Can. J. Biochem. Physiol..

[76] Rouser G., Fleischer S., Yamamoto A. (1970). (1970) two dimensional then layer chromatographic separation of polar lipids and determination of phospholipids by phosphorus analysis of spots. Lipids.

[77] Perez-Riverol Y., Csordas A., Bai J., Bernal-Llinares M., Hewapathirana S., Kundu D.J., Inuganti A., Griss J., Mayer G., Eisenacher M., Pérez E., Uszkoreit J., Pfeuffer J., Sachsenberg T., Yilmaz S. (2019). The PRIDE database and related tools and resources in 2019: Improving support for quantification data. Nucleic Acids Res..

[78] Keiser T.L., Azad A.K., Guirado E., Bonacci R., Schlesinger L.S. (2011). Comparative transcriptional study of the putative mannose donor biosynthesis genes in virulent *Mycobacterium tuberculosis* and attenuated *Mycobacterium bovis* BCG strains. Infect Immun..

