# Supplementary material for: Mycobacterium tuberculosis encodes a YhhN family membrane protein with lysoplasmalogenase activity that protects against toxic host lysolipids
Source: J Biol Chem. 2022 Mar 18;298(5):101849. doi: 10.1016/j.jbc.2022.101849 (PMC9052158; doi:10.1016/j.jbc.2022.101849)
Supplement: Supplemental Figures S1–S5, Tables S1 and S2 [file mmc1.docx]

Revised Supporting information (20220317)

***Mycobacterium tuberculosis* encodes a YhhN family membrane protein with lysoplasmalogenase activity that protects against toxic host lysolipids**

Marianne S**.** Jurkowitz*^a^, Abul K. Azad*^b^, Paula C. Monsma^c^, Tracy L Keiser^d^, Jean Kanyo^e^, TuKiet T. Lam^e,f^, Charles E. Bell^a,g^, and Larry S. Schlesinger^b^

^a^Department of Biological Chemistry and Pharmacology, The Ohio State University, Columbus, Ohio, 43210, ^b^Texas Biomedical Research Institute, 8715 W. Military Drive, San Antonio, TX, 78227, ^c^Department of Neuroscience, Ohio State University, Columbus, Ohio 43210, ^d^Department of Moleculaire Microbiologie, Vrije Universiteit, De Boelelaan1105,1081 HV Amsterdam, Netherlands, , ^e^Keck MS & Proteomics Resource, Yale University, New Haven, CT, ^f^Department of Molecular Biophysics and Biochemistry, Yale University, New Haven, CT, ^g^Department of Chemistry and Biochemistry, The Ohio State University, Columbus, Ohio 43210,

**Figure S1 Mass Spectrometry/ protein identification of gel bands 23 kD (S1A) and 38 kD (S1B) of gel shown in Figure 3 of manuscript.**

**Figure S2 PAGE gel of the partial purification of MtbYhhN 200aa**

**Figure S3 Lysoplasmenylcholine (pLPC) and lysoplasmenylethanolamine (pLPE) cause ATP depletion in human macrophages.**

**Figure S4 The level of mycobacterial association with human macrophages during early hours of infection between empty vector control and Rv1401-expressing *M.smeg* clones is not significantly different**

**Figure S5.** **Topologies of MtbYhhN proteins predicted using the Transmembrane Hidden Markov Model (TMHMM).**

**Table S1 Stoichiometry: Quantitative correlation between moles of substrate disappearance and moles of products formed in the MtbYhhN lysoplasmalogenase reaction**

**Table S2 Primers used for cloning the *M.tb* gene Rv1401 into plasmid vector for overexpressing in *M.smeg***

**Figure S1A Protein Identification: Gel band at about 23 kD, Figure 3 in manuscript**


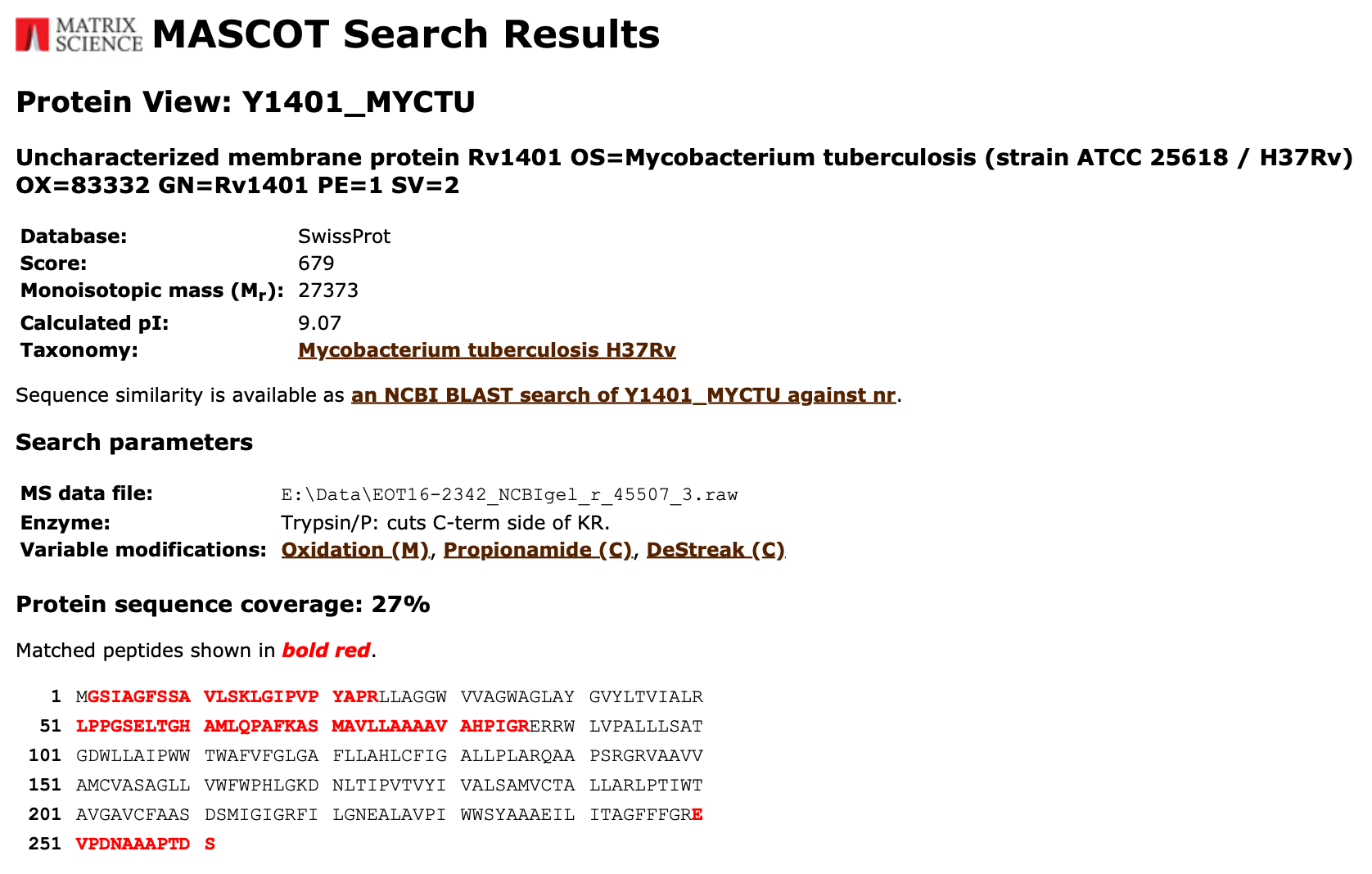


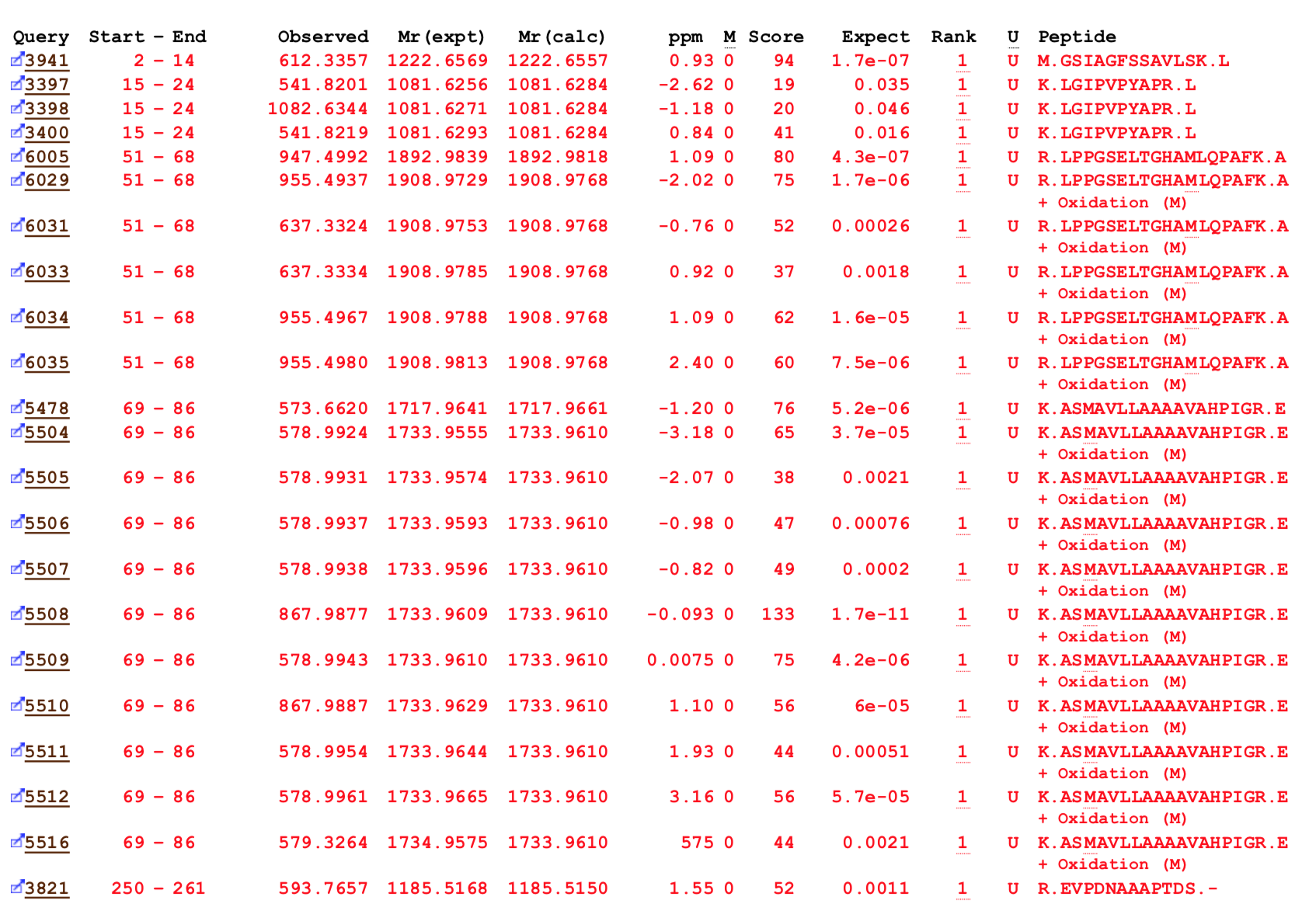


**Figure S1B Protein Identification: Gel band at about 38 kD, Figure 3 in manuscript**


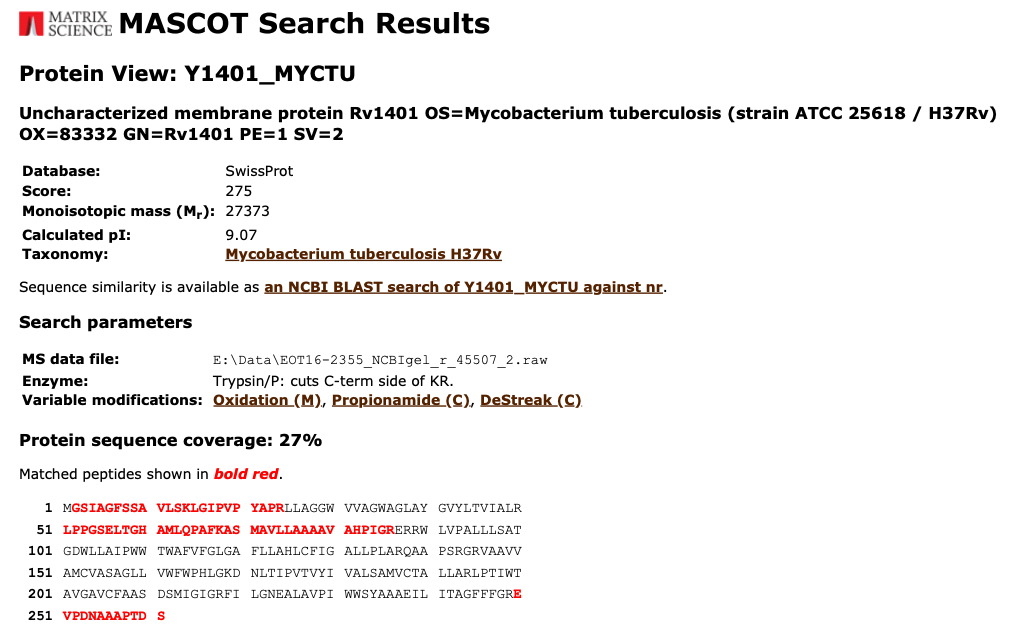


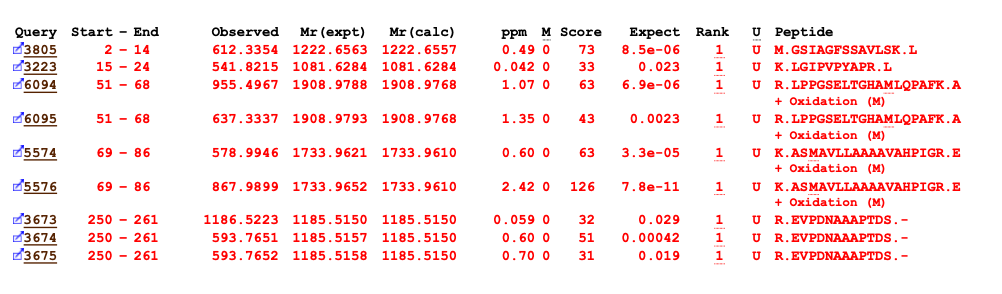


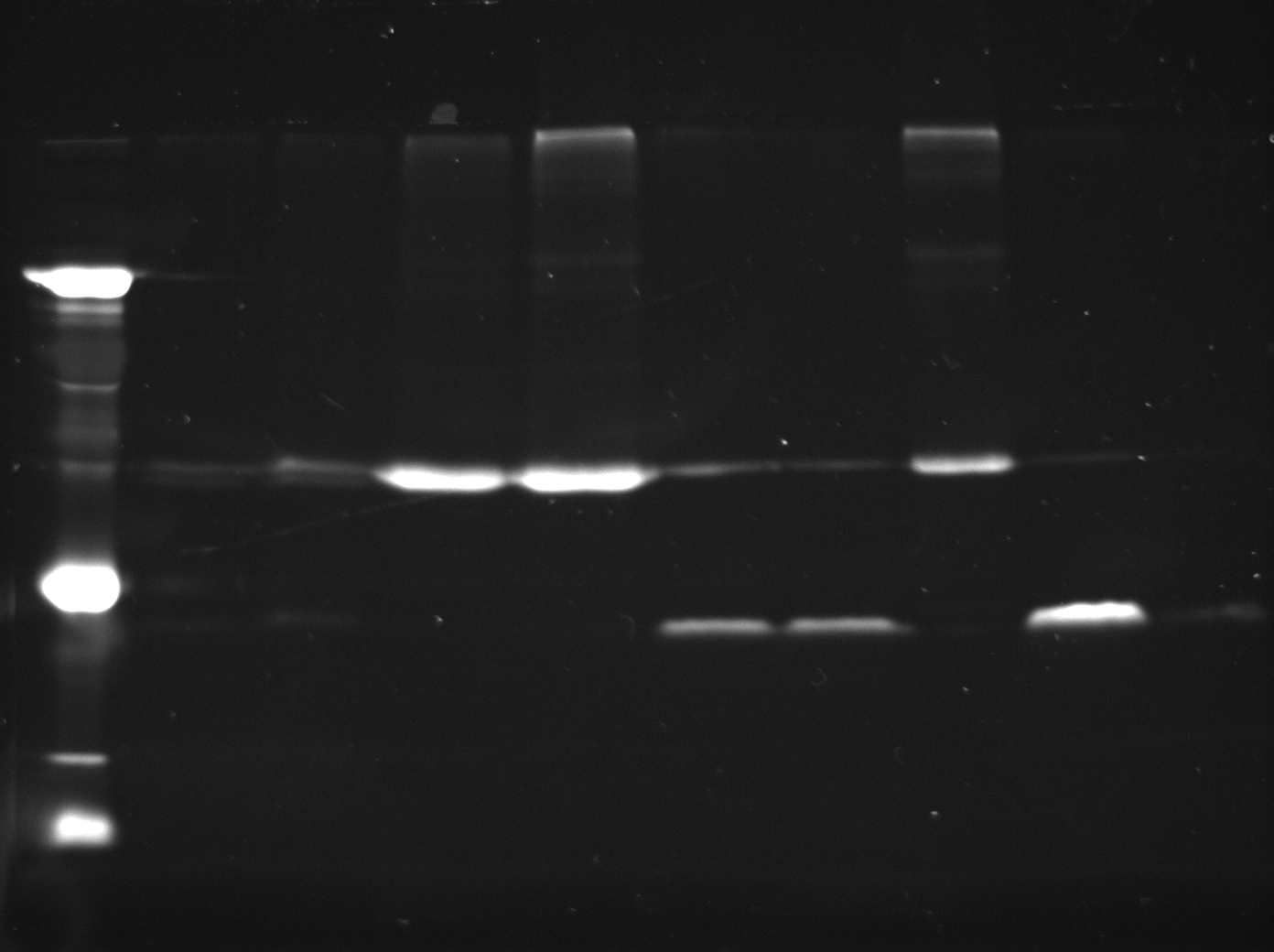

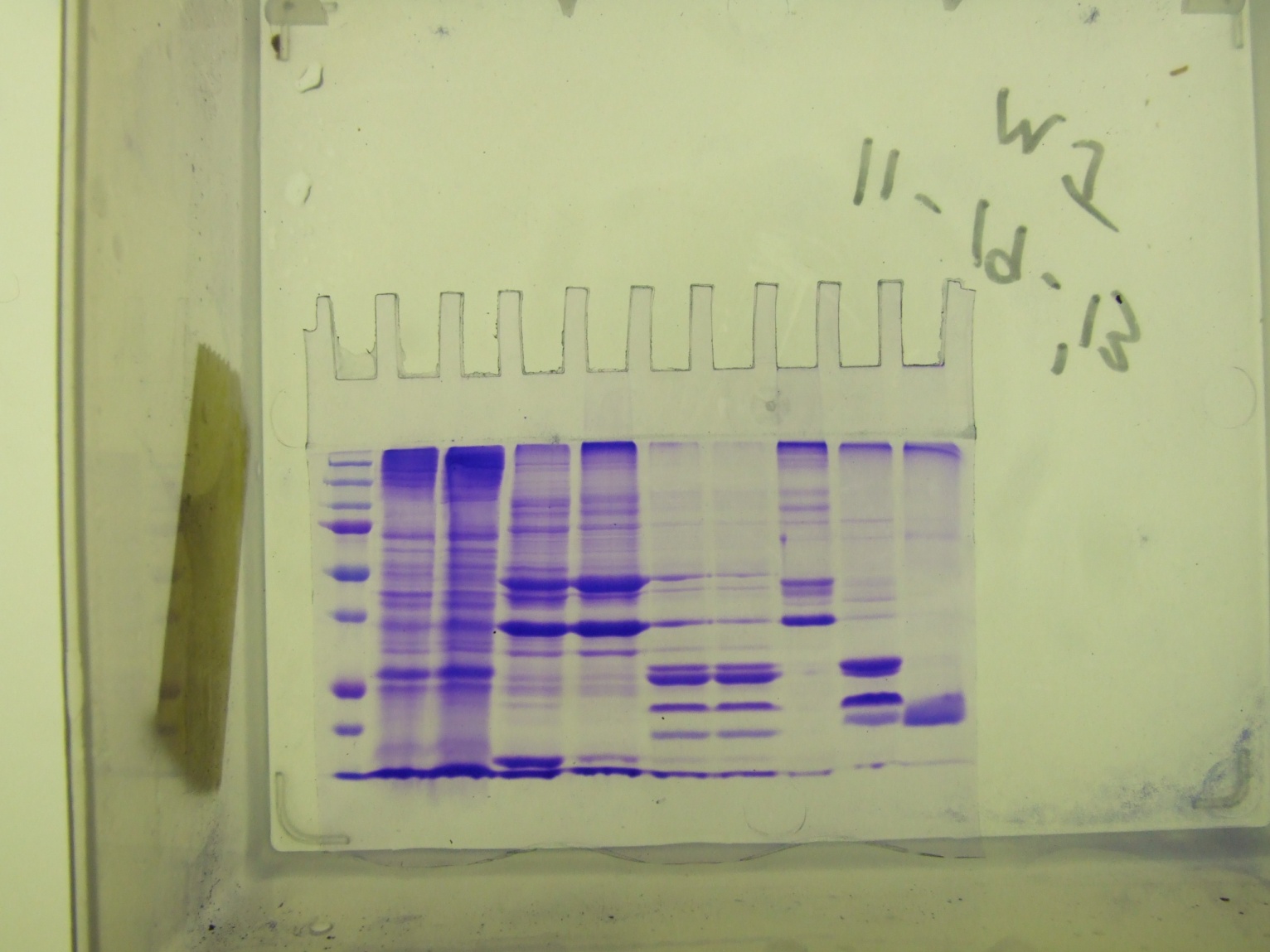


10

20

25

37

50

75

100

250

1

5

3

4

2

7

6

3

6

7

5

4

2

1

**Figure S2**. **The partial purification of MtbYhhN 200aa**

The Rv1401 protein of 200 amino acids was cloned and expressed in *E. coli* as a GFP-His-fusion protein. The gel is a Laemmli 11%. The fluorescence image (right) was obtained before staining with Coomassie (left). Lanes: 1, MW standards; 2, DDM treated *E. coli* membrane fraction; 3, 100 K supernatant put on to Nickel-affinity column; 4, Nickel-affinity column fraction #11; 5, Nickel-affinity column fraction #12; 6, TEV treated fraction #12 (concentrated); 7, duplicate of lane 6. In lanes 4 and 5 the dark bands at 36 kDa are the MtbYhhN-GFP-fusion protein, which is strongly fluorescent. Lanes 6 and 7 are the fractions obtained after TEV treatment of the fusion protein. In the Coomassie stained gel, the black arrow points to a band we identified as the purified 200 amino acid MtbYhhN protein (19 kDa)., the band above the MtbYhhN protein at about 23 kDa is GFP (blue arrow), and is seen as a fluorescence band in image on the right. The thick band above GFP at 26 kDa (blue arrowhead) is the TEV protein.

| 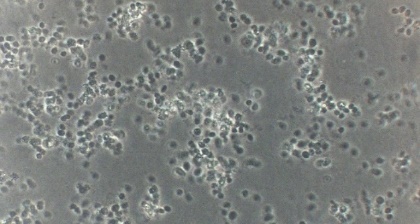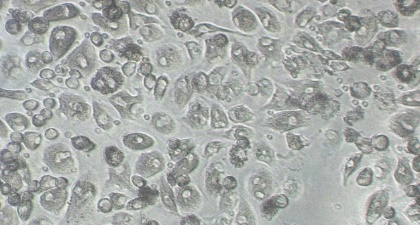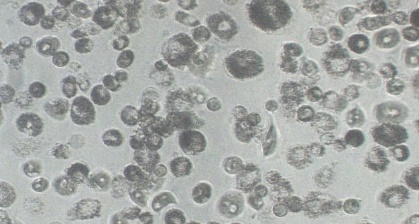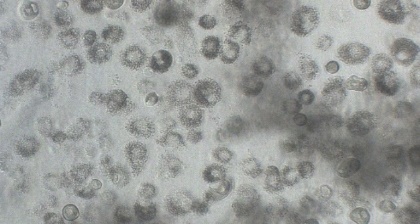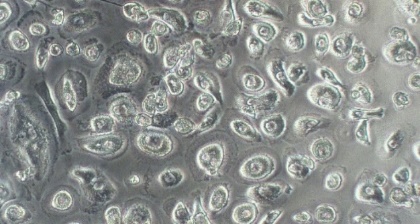 Control No addition  1% triton  pLPC  μM pLPC : 50 100 200 |
| --- |
| μM pLPE: 100 200 400  1% triton 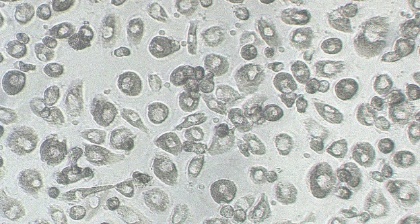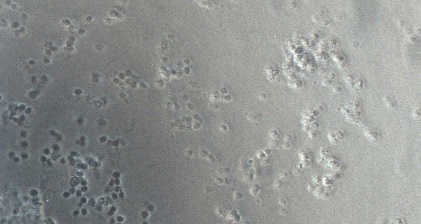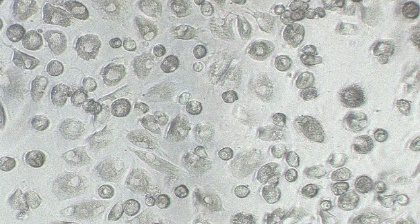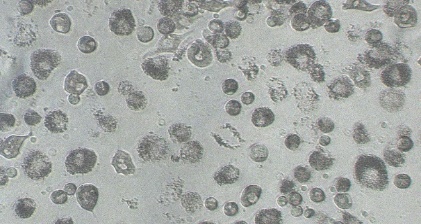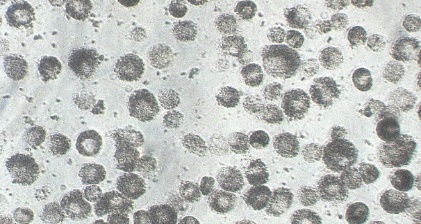 pLPE  Control No addition |

**Figure S3. The effects of lysoplasmenylcholine (pLPC) and lysoplasmenylethanolamine (pLPE) on human macrophages.** The lysophospholipids were added to macrophage cultures in RPMI+HEPES media and intracellular ATP levels were measured at 1, 2, and 3 h post-addition of lysophospholipids. The macrophage toxicity data are shown from pLPC (upper 6 panels) and pLPE (lower 6 panels) experiments. Following ATP assay experiments, the cell morphology was assessed using phase contrast microscopy at 3.5 h post-treatment. The control represents macrophages with no lysolipid added (black circles). The Triton control macrophages were treated with 1% triton (purple squares). The macrophages in other plates were treated with designated amounts of either pLPC or pLPE: brown squares, 50 μM; green triangles, 100 μM; blue diamonds, 200 μM; red diamonds, 400 μM. The points are averages of duplicate plates and error bars are S.E.M. The scale bars on the photomicrographs represent 100 μm (micrometers). This experiment is representative of two separate experiments with different cell cultures.

**
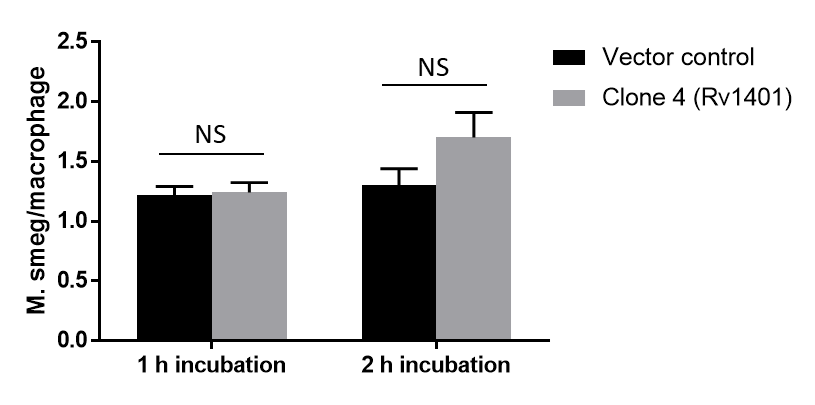
**

**Figure S4. Association of the mc2155/SMT3 (vector control) and of the mc2155/SMT3Rv1401(1-261) *M.smeg* strains with human macrophages.** MDMs cultured on coverslips in duplicate for each time point were infected with either *M.smeg* empty vector control or Rv1401-expressing cells at an MOI of 5. After 1 or 2 h infection, the macrophage monolayer was washed, fixed with 4% paraformaldehyde, and then subjected to auramine-rhodamine staining. Coverslips from each time point were examined by fluorescence microscopy, counting the stained red bacteria associated with each macrophage. Approximately 100 macrophages were examined for each type of *M.smeg* strain and the data were expressed the number of *M.smeg*/macrophage. Shown is a representative experiment of N = 2 independent experiments. P = NS (non-significant).

| 200aa | 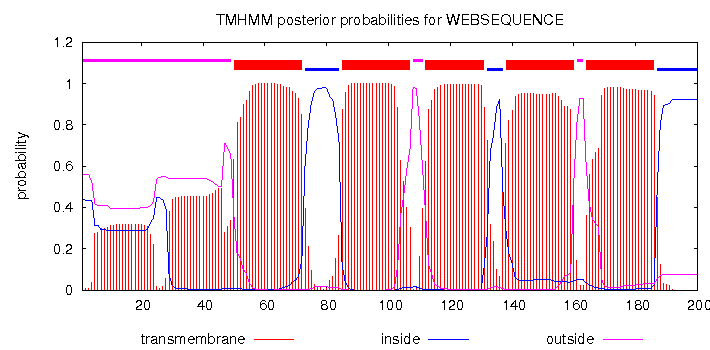 |
| --- | --- |
| 231 aa | 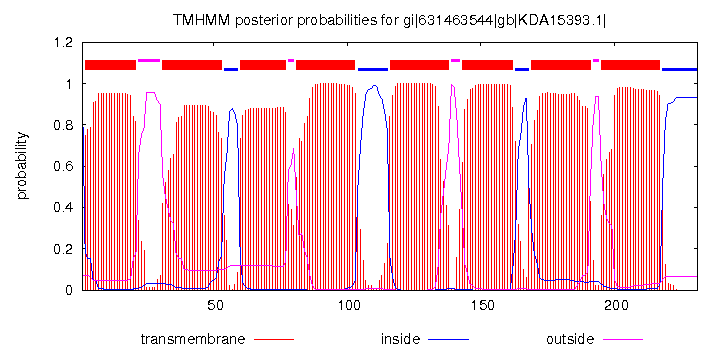 |
| 237 aa | 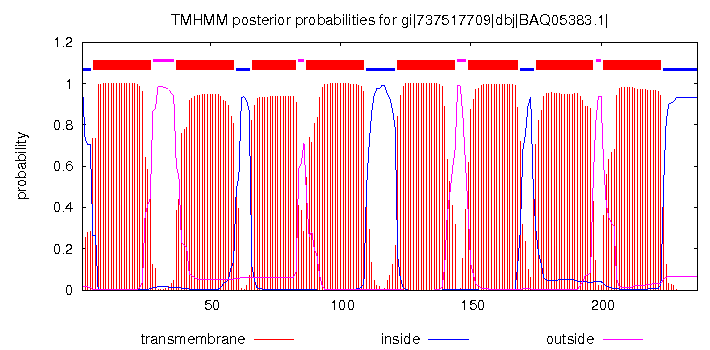 |
| 261 aa | 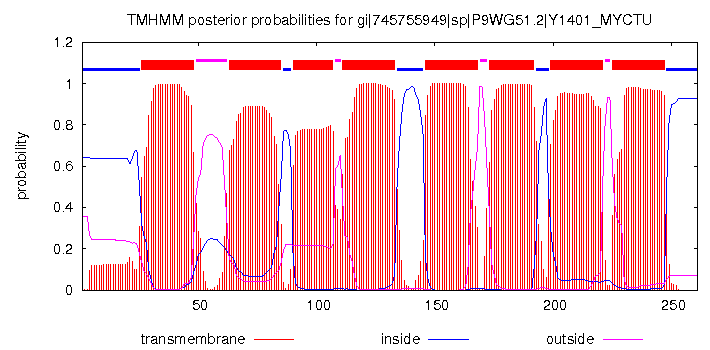  Residue number in amino acid chain |

**Figure S5. Topologies of MtbYhhN proteins predicted using the Transmembrane Hidden Markov Model (TMHMM).** The X- axis shows the residue number in the amino acid chain from the N-terminus to the C terminus ending at residue 261.

The plots are aligned with their C-terminals at amino acid 261 to convey the fact that the three smaller proteins are with N-terminal truncations,

The Y- axis shows the probability of transmembrane location at each residue position, as indicated by the thin red lines (vertical). The cartoon along the top of the plot indicates predicted TM alpha helices (thick red lines), and cytoplasmic (blue) or periplasmic (majenta) loops. (45).

**Table S1.**

**Stoichiometry: Quantitative correlation between moles of substrate disappearance and moles of products formed in the MtbYhhN lysoplasmalogenase reaction**

| Time (min) | Substrate disappearance and product formation (nmoles/3 ml) | | |
| --- | --- | --- | --- |
|  | Lysoplasmenylethanolamine disappearance ^b^ | Aldehyde production ^a^ | Glycerophospho-ethanolamine production ^b^ |
| 0 | 980 ± 57 | 0 | 0 |
| 10 | 360 ± 51 | 723 ±48 | 630 ± 48 |

Incubation conditions: Hydroxyapatite column fraction 10 μg; 1050 nmoles lysoplasmenylethanolamine substrate (350 μM), 70 mM glycylglycine buffer pH 7.0, final volume 3.0 ml.

^a^ Enzyme assay 1 (coupled enzyme assay)

**^b^** Enzyme assay 2 (lipid extraction, followed by separation of lipids and aqueous fractions, and products identified and quantified using TLC and phosphorus assays, as described in the Methods section.) The reactions were run in duplicates, and reported as the mean +/- S.D.

**TABLE S2.**

**Primers used for cloning the *M.tb* gene Rv1401 into plasmid vector for overexpressing in *M.smeg***

| Protein Name | Accession number | *M. tuberculosis* strain | Amino Acids | Forward Primer  (using BamHI site) | Reverse Primer  (using EcoRV site) |
| --- | --- | --- | --- | --- | --- |
| Membrane protein RV1401 | NP_  215917.1 | H_37_R_v_ | 200 | 5’-gcc gga tcc cat gtt gca gcc cgc gtt caa g-3’ | 5’-gcg cga tat cct agc tat ccg tag gtg ctg cgg c -3’ |
| YhhN family membrane protein | GenBank  KDA15393.1 | C2 | 231 | 5’-gcc gga tcc cgt ggt cgc cgg ctg ggc ggg c-3’ | 5’-gcg cga tat cct agc tat ccg tag gtg ctg cgg c -3’ |
| Hypothetical protein | GenBank: BAQ05383.1 | KURONO_1582 | 237 | 5’-gcc gga tcc ctt gct ggc cgg cgg ctg ggt gg-3’ | 5’-gcg cga tat cct agc tat ccg tag gtg ctg cgg c -3’ |
| Uncharacterized membrane protein Rv1401 | UniProtKB/  Swiss-Prot:  P9WG51.2 | H_37_R_v_ | 261 | 5’-gcc gga tcc cat ggg gtc gat cgc ggg ttt ctc g-3’ | 5’-gcg cga tat cct agc tat ccg tag gtg ctg cgg c -3’ |
